# Long‐term clinical benefits of periodontal interventions in strict supportive periodontal care: A systematic review

**DOI:** 10.1002/jper.70027

**Published:** 2025-11-18

**Authors:** Varkha Rattu, Tishani Patel, Jasmine Loke, Hari Petsos, Luigi Nibali

**Affiliations:** ^1^ Periodontology Unit Centre for Host‐Microbiome Interactions Dental Institute, King's College London London UK; ^2^ Department of Periodontology Goethe University Frankfurt/Main Centre of Dentistry and Oral Medicine (Carolinum) Frankfurt am Main Germany

**Keywords:** disease management, periodontitis, tooth loss

## Abstract

**Background:**

Periodontitis requires long‐term management to prevent recurrence and tooth loss. While active periodontal therapy (APT) aims to reduce probing pocket depth (PPD) and improve clinical attachment level (CAL), strict supportive periodontal care (SPC) is essential for maintaining these clinical outcomes. This study systematically reviewed the long‐term benefits of APT interventions (test groups) compared to standard control interventions (control groups) in patients undergoing SPC as part of randomized controlled trials (RCTs).

**Methods:**

A systematic search of databases and journals identified RCTs with a minimum follow‐up of 10 years. Studies comparing APT interventions, such as regenerative techniques, to standard care in patients adhering to SPC were included. Primary outcomes assessed included tooth loss, and secondary outcomes included PPD reduction, CAL gain, and patient‐reported outcome measures (PROMs). Descriptive analyses were undertaken on all studies, and meta‐analyses were conducted to analyze the weighted mean differences (WMDs) for PPD, CAL, and tooth loss between test and control interventions when appropriate.

**Results:**

From an initial screening of 4582 articles, 9 were deemed suitable and included for descriptive analyses. Four publications of 3 studies were included in meta‐analyses comparing guided tissue regeneration (GTR) and open flap debridement (OFD) over a 10‐to 20‐year SPC follow‐ups. Regenerative techniques demonstrated significant CAL gains at 1‐year follow‐up compared to control groups. At 10 to 20 years, PPD and CAL outcomes were comparable between groups. Tooth loss was minimal across all groups. Variability in SPC protocols and operator experience may have influenced outcomes.

**Conclusions:**

Long‐term periodontal stability does not appear to be affected by the choice of initial intervention in patients who adhere to a rigorous SPC program. While regenerative techniques offer short‐term advantages, their long‐term benefits may reduce in comparison to non‐regenerative methods under SPC. Future research should focus on standardized SPC protocols and cost‐effectiveness to optimize periodontal care.

**Plain Language Summary:**

Periodontitis is a chronic immune‐inflammatory condition that can lead to increased risk of tooth loss if not managed. Treatment such as non‐surgical periodontal therapy (NSPT), adjunctive therapies, or various surgeries can improve periodontal health. This systematic review examined randomized controlled trials (RCTs) where patients received different periodontal treatments and were then followed up for ≥10 years while attending maintenance visits. Periodontal surrogate and true endpoints were compared between the test and control groups. The findings suggest that long‐term outcomes were similar between groups, provided patients remained in consistent maintenance care. This highlights that the key to long‐term success is not necessarily which active treatment is used, but possibly the adherence to structured maintenance. These results allow clinicians and researchers to understand the types of treatments that may have lasting benefits and reinforces the importance of long‐term care in preventing disease progression.

## 1 INTRODUCTION

The long‐term management of periodontitis remains a critical concern in contemporary clinical practice given the chronic nature of the disease and the inherent risk of recurrence. While active periodontal therapy (APT) aims to achieve clinical improvements through probing pocket depth (PPD) reduction and clinical attachment level (CAL) gain, the longevity of these outcomes over extended periods, particularly under supportive periodontal care (SPC) remains insufficiently elucidated. Furthermore, tooth loss, the true endpoint of periodontitis, must be evaluated in the context of long‐term maintenance to assess the true therapeutic benefit. Patient‐reported outcome measures (PROMs), though essential in capturing functional and psychosocial dimensions of care, are often underrepresented in the existing literature.

APT encompasses a range of interventions from non‐surgical periodontal therapy (NSPT) with or without the use of adjunctive therapies, to open flap debridement (OFD), resective and regenerative surgical techniques.^1^ According to European Federation of Periodontology (EFP) guidelines, SPC is recommended once patients achieve specific clinical endpoints, such as the absence of PPD ≥6 mm and no bleeding at probing (BoP) at 5 mm sites.^1^, ^2^ However, the optimal criteria for initiating SPC remains a subject of debate, with some authors proposing alternative endpoints.^3^ Regardless of the specific thresholds, SPC should begin promptly following successful completion of APT.

With a recent systematic review indicating only 11%–27.6% of periodontitis patients attain the EFP‐proposed therapeutic endpoints following APT, long‐term SPC has shown remarkable value with only 3.14% of all teeth lost, underscoring SPC's value in preventing long‐term disease progression and associated tooth‐loss.^4^ Therefore, evaluating the durability of clinical improvements through various interventions in the context of SPC is essential to guide treatment planning and optimize long‐term care strategies.

While landmark research studies have shown that surgical periodontal therapy and the use of adjunctive systemic antimicrobials yield superior short‐term clinical outcomes compared to NSPT, there is limited robust evidence on the sustained benefits of these interventions over long‐term rigorous SPC.^5^, ^6^, ^7^, ^8^ By systematically reviewing the available literature, this review seeks to address whether any specific periodontal interventions offer sustained clinical benefits, beyond standard care, during long‐term SPC. For this systematic review, standard care refers to the control interventions provided in the comparator arms of the included RCTs, typically compromising conventional periodontal treatments such as NSPT, OFD, or regenerative procedures using established biomaterials. While the specific definition varied among studies, this review sought to determine whether long‐term (≥10 years) SPC influences the sustainability of clinical outcomes between test and control interventions. The findings aim to enhance understanding of the long‐term efficacy APT interventions, guiding clinicians in selecting interventions that support lifelong periodontal stability. This led to the following focused question:

- *Focused question (FQ)*: Are the clinical benefits of periodontal interventions during APT compared with the standard control intervention maintained long‐term (≥ 10 years) in patients undergoing strict supportive care?

APT will refer to any or all interventions aimed at resolving active inflammation and reducing PPD including, but not limited to, those outlined in the EFP S3 treatment guidelines.^1^ Strict SPC was defined as a structured program delivered at regular intervals, typically every 3–12 months as advised by the treating clinician, following APT completion.

## 2 MATERIALS AND METHODS

### 2.1 Protocol development and registration

A systematic review protocol was developed in accordance with the Preferred Reporting Items for Systematic Reviews and Meta‐Analyses (PRISMA) guidelines.^9^ The protocol details were registered with PROSPERO on December 1, 2023 (ID: CRD42023487207).

### 2.2 Eligibility criteria

**PICOS components**

*Population*: Adult human patients with periodontitis (excluding as manifestation of systemic or necrotizing diseases), who have completed APT and undergone SPC visits for ≥10 years.
*Intervention*: APT encompasses many interventions ranging from behavioral changes to surgical interventions.^1^ The intervention group was defined as those delivered in the test arm of each included RCT.
*Comparison*: Standard care was defined as the control intervention provided during APT in each included study. The included interventions reflect established clinical protocols routinely used in periodontal practice.
*Outcome measures*:
  - Primary outcome for FQ assesses the incidence of tooth loss.
  - Secondary outcome for FQ assesses PPD reduction, CAL gain, PROMs of each intervention.
*Study design*: Randomized controlled trials (RCTs) with a minimum SPC follow-up of 10 years.

### 2.3 Literature search

A comprehensive search strategy, without language restrictions, was devised in collaboration with an experienced librarian, incorporating both MeSH terms and free‐text keywords (Supplemental Material 1 in the online *Journal of Periodontology*). The electronic databases searched included Ovid MEDLINE, Ovid EMBASE, the Cochrane Central Register of Controlled Trials (CENTRAL), covering the period from 1947 to July 22, 2025. Manual searches were conducted in the *Journal of Dental Research, Journal of Clinical Periodontology, Journal of Periodontology*, and *Journal of Periodontal Research* for the same period. Reference lists of the included articles and pertinent reviews were also examined manually. All records retrieved from databases were exported into reference management software (EndNote 20), where automatic detection and manual verification based on titles, authors and publication details was performed.

### 2.4 Screening and study inclusion

Study selection was conducted independently by 2 independent reviewers using a 2‐step process: (i) screening of titles and abstracts (authors T.P. and J.L.); followed by (ii) full‐text analysis, with reasons for exclusion (authors V.R. and T.P.) (Supplemental Material 2 in the online *Journal of Periodontology*). Full texts were obtained for those studies selected by at least 1 reviewer. Disagreements were resolved by consensus. An arbitrator (author L.N.) was consulted if the disagreement could not be resolved. Inter‐observer agreement for both stages was evaluated via the Cohen's kappa statistic.

### 2.5 Data collection

#### 2.5.1 Data extraction

Data extraction was undertaken by 1 reviewer (V.R.) and independently verified by another (T.P.). It included general study and population characteristics from journal articles (Table 1). Clinical information, including tooth type and defects treated, types of APT in the control and intervention groups, SPC regimen, incidence of tooth loss, PPD and CAL changes, and PROMs were extracted (Table 2). Extracted data were entered into tables using Microsoft Excel and stratified based on the different interventions included in the study. Where differences in parameters were not recorded, differences in means and standard deviation (SD) were calculated using the formula for independent patient samples as paired individual patient data (IPD) were not available.

**TABLE 1:** Study characteristics of included studies.

										APT			
Reference	Funding	Country	Setting	Patient sample size: Baseline Final	Demographics	Medical history of significance	Smoking history	Diagnosis and severity of periodontitis	Tooth types and defects included in the RCT	All participants	Control Test	SPC regimen and duration	SPC compliance
**Comparison of regenerative surgery and OFD in the treatment of residual sites associated with infrabony defects**													
^10^	3, 4, 5	Germany	2	16 15	Age range: 29 – 61 years Sex F/M: 8/8	Generally healthy	6 former; 4 current; 5 never	Severe ChP^11^	44 intra‐bony defects included (NB: 41 defects at 10 years)	Quadrant‐wise scaling (without adjunctive therapies)	OFD GTR using bioabsorbable polyactide acetyltributyl citrate barrier^*^ ^,^ ^†^	Duration: 10 ± 1 years Frequency: ‐ 3‐monthly for first year at university hospital	3 patients > /1 SPC/year 12 patients < 1 SPC/year
^12^	3, 5	Germany	2	16 12	(Age range: 29 – 61 years) Sex F/M: 8/8	Generally healthy	5 former; 2 active; 5 never	Severe ChP^11^	38 infrabony defects included with PPD ≥5 mm	Quadrant‐wise scaling (without adjunctive therapies)	OFD GTR using bioabsorbable polyactide acetyltributyl citrate barrier^*^	Duration: 20 ± 1 year Frequency: ‐ 3‐monthly for first year at university hospital ‐ ≥1 SPC/ year with GDP (for first 10 years	<1 SPC/ year (for most patients from 10‐20 years)
^13^	5	Germany	2	56 38	Mean age: 52 ± 12.6 years	No systemic diseases that could interfere with healing	1 smoker in EMD and EMD + GTR groups; 0 in other groups	Not reported	Intra‐bony defect with PPD ≥ 6 mm and an intra‐bony component ≥3 mm as detected on the radiographs	OHI SRP	OFD Group 1: Regen using EMD^†^ Group 2: Regen with GTR^‡^ Group 3: Combination – EMD + GTR^†^ ^,^ ^‡^	Duration: 10 years Frequency: ‐ Fortnightly for first 2 months ‐ Monthly for first year ‐ 3‐monthly thereafter Consists of: ‐ OHI ‐ PMPR	All included patients at re‐assessment complied with 3‐monthly SPC
^14^	1, 3	Italy	1	45 41	Mean age: 42.8 ± 8.9 years (range: 25‐61 years) Sex F/M: 24/21	Good general health	Current: 6 (smoked < 20 cigarettes/ day) ‐ 2 in each group	Not reported	Incisors: 14 Cuspids: 6 Bicuspids: 13 Molars: 12 Defect: 1 deep intra‐bony defect in the interproximal area + does not extend into a furcation	Not reported	Access flap (MWF) Group 1: Titanium‐reinforced e‐PTFE^§^ membranes + MPPT Group 2: Access flap + e‐PTFE membrane^¶^	Duration: 20 yearsFrequency: ‐ Monthly for first year ‐ 3‐monthly thereafter	All included patients at re‐assessment complied with 3‐monthly SPC
**Comparison of biomaterials used in regenerative surgery in the treatment of residual sites associated with infrabony defects**													
^15^	1, 3	Italy	1	45 42	Mean age: 49.9 ± 9.8 years (range: 28 – 71 years) Sex F/M: 21/24	Good general health	5 smokers		Incisors: 14 Cuspids: 6 Bicuspids: 13 Molars: 12 Defect: 1 deep intra‐bony defect in the interproximal area + does not extend into a furcation	OHI SRP Pocket elimination flap surgery (different sites to experimental site)	M‐MIST Group 1: M‐MIST + EMD^†^ ^,^ ^#^ Group 2: M‐MIST + BMDX	Duration: 10 years Frequency: ‐ 3‐monthly	3.2 ± 0.3 visits/ year Group 1: 3.0 ± 0.2 visits/ year Group 2: 3.2 ± 0.3 visits/ year
^16^	Original study – 3 This study – 1	Norway	1	40 26	Mean age: 53 years (range: 42 – 67 years) Sex F/M: 20/20	Systemically healthy	Non‐smokers	ChP^11^	Defect: ≥1 defect with PPD ≥7 mm with interproximal intra‐bony defect with a depth ≥5 mm		Regen with autogenous BG alone Regen with autogenous BG + GTR using a chair‐side‐prepared bioresorbable polylactic acid barrier^∥^ extending 3 mm over the defect margins to cover the bone graft.	Duration: 10 years Frequency: ‐ 3‐monthly for first 9 months ‐ 3‐6‐monthly thereafter Consisted of: ‐ OHI ‐ Supra‐ and subgingival biofilm control ‐ Fluoride application	N/A
^17^	5	Hungary	2	24 22	Age range: 34 – 67 years Sex F/M: 16/8	No systemic diseases of concern	Not reported	Gen advanced ChP^11^	Anterior teeth: 10 Premolars: 9 Molars: 3 Defect: 1 intra‐bony defect with a PPD ≥6 mm, and an intrabony component of ≥4 mm		Regen with EMD + β‐TCP Regen with EMD + NBM^†^ ^,^ ^#^ ^,^ ^**^	Duration: 10 years Frequency: ‐ Weekly for first 6 months ‐ 1‐monthly for subsequent 6 months ‐ 3‐6 monthly thereafter Consisted of: ‐ OHI ‐ Supra + subgingival instrumentation ‐ ± occlusal adjustment	≥1 visit/ year
**Comparison of different surgical therapies in the treatment of residual sites not associated with infrabony defects**													
^18^		Italy	1	25	Mean age: 45.2 years Sex F/M: 15/10	Non‐contributory MH or medications	≤5 cigarettes/ day	Moderate to advanced	Teeth with ≥1 sites with PPD ≥5 mm	OHI SRP Re‐assessment after 3‐weeks	MWF CAF + Adjunctive use of CO_2_ laser	Duration: 15 years Frequency: ‐ Fortnightly for first 3 months ‐ x2/ year thereafter Consisted of: ‐ Supra + subgingival instrumentation	N/A
**Comparison of surgical and non‐surgical therapies in the treatment of residual sites not associated with infrabony defects**													
^19^	3	Sweden	2	64 45	Control: 43.8 ± 8.9 years Test: 45.6 ± 7.3 years	Not reported			≥12 non‐molar teeth with deep PPD ≥6 mm with ≥6 mm alveolar bone loss	OHI	SRP (4‐6 sessions within 2 months using USS device) MWF (4‐6 sessions within 2 months)	Duration: 13 years Frequency: ‐ 3‐4 sessions/ year Consists of: ‐ OHI Subgingival instrumentation of residual sites under LA	N/A

Codes: Funding – 1 (None), 2 (Government), 3 (Private), 4 (Self‐funded), 5 (University); Setting – 1 (Private practice), 2 (University hospital).

Abbreviations: AgP, aggressive periodontitis; BG, bone graft; BMDX, bone mineral derived xenograft; CAF, coronally advanced flap; ChP, chronic periodontitis; EMD, enamel matrix derivative; Gen, generalized; GTR, guided tissue regeneration; LA, local anesthetic; Loc, localized; M‐MIST, modified minimally invasive surgical therapy; MPPT, modified papilla preservation technique; MWF, modified Widman flap; NBM, natural bone mineral; NSPT, non‐surgical periodontal therapy; OFD, open flap debridement; OHI, oral hygiene instructions; PMPR, professional mechanical plaque removal; Regen, regenerative surgery; SE, socio‐economics; SRP, scaling and root planing; USS, ultrasonic; β‐TCP, β‐tricalcium phosphate.

^*^ Resorbable membrane: Guidor Matrix Barrier, bioabsorbable polylactide acetyl tributyl citrate barriers, Guidor AB, Huddinge, Sweden (used in studies 10, 12).

^†^ Enamel matrix derivatives: Emdogain, Straumann, Basel, Switzerland (used in studies 10, 13, 15, and 17).

^‡^ Resorbable membrane: Resolut (expanded polytetrafluoroethylene), W.L. Gore & Associates, Flagstaff, AZ, USA (used in study 13).

^§^ Non‐resorbable membrane: expanded polytetrafluoroethylene (ePTFE) membrane; manufacturer not specified (used in study 14).

^¶^ Non‐resorbable membrane: titanium‐reinforced dense polytetrafluoroethylene (dPTFE) membrane, manufacturer not specified (used in study 14).

^#^ Bone‐mineral‐derived xenograph, Bio‐Oss, Geistlich, Wolhusen, Switzerland (used in studies 15 and 17).

^∥^ Resorbable membrane: chairside‐prepared bioresorbable polylactic acid barrier (Atrisorb, Atrix Laboratories Inc., Fort Collins, CO, USA) (used in study 16).

^**^ β‐tricalcium phosphate (β‐TCP): Cerasorb, Curasan Pharma, Kleinostheim, Germany (used in study 17).

**TABLE 2:** Clinical findings of included studies.

Reference	APT Control (*n* = no. of patients) Test (*n* = no. of patients)	Tooth‐loss	PPD baseline (mm)	PPD at 1 year (^*^unless otherwise stated) (mm)	PPD Final (mm)	PPD change from baseline to 1 year (^*^unless otherwise stated) (mm)	PPD change from 1 year to final (^*^unless otherwise stated) (mm)	PPD change from baseline to final SPC (mm)
**Comparison of regenerative surgery and OFD in the treatment of residual sites associated with infrabony defects**								
^10^	OFD (*n* = 15)	2	8.77 ± 2.22	5.06 ± 2.30	4.35 ± 1.22	−3.71 ± 2.85	−0.71 ± 2.49	−4.41 ± 2.37
	b. GTR using bioabsorbable polyactide acetyltributyl citrate barrier (*n* = 15)^*^	4	8.69 ± 1.92	4.56 ± 1.65	4.44 ± 1.42	−4.14 ± 2.00	−0.11 ± 1.91	−4.25 ± 2.44
^12^	OFD (*n* = 12)	3	8.75 ± 2.33	5.06 ± 2.38	4.16 ± 1.38	−3.69 ± 2.13	−0.94 ± 0.66	−4.59 ± 3.25
	b. GTR using bioabsorbable polyactide acetyltributyl citrate barrier (*n* = 12)^*^	4	8.70 ± 2.03	4.40 ± 1.68	4.93 ± 1.87	−4.30 ± 3.04	−0.53 + 0.38	−3.77 ± 2.66
^13^	OFD (*n* = 9)	Not reported	8.6 ± 1.5	4.9 ± 1.8	5.1 ± 1.2	−3.7 ± 2.34	+0.2 ± 2.16	−3.5 ± 1.92
	b. Group 1: Regen using EMD (*n* = 10)		8.4 ± 1.9	4.3 ± 1.2	4.8 ± 1.1	−4.1 ± 2.25	+0.5 ± 1.63	−3.6 ± 2.2
	Group 2: Regen with GTR (using Revolut) (*n* = 10)		8.4 ± 1.7	4.2 ± 1.3	5.0 ± 1.0	−4.2 ± 2.14	+0.8 ± 1.64	−3.4 ± 1.97
	Group 3: Combination – EMD + GTR (*n* = 9)		8.6 ± 1.5	4.3 ± 1.3	5.1 ± 1.2	−4.3 ± 1.98	+0.8 ± 1.77	−3.5 ± 1.92
^14^	Access flap (MWF) (*n* = 15)	2	8.3 ± 2.0	3.7 ± 1.3	5.5 ± 2.7	−4.6 ± 2.39	+1.9 ± 0.6	−2.8 ± 3.36
	b. Group 1: Titanium‐reinforced e‐PTFE membranes + MPPT (*n* = 15)	0	8.4 ± 2.5	2.1 ± 0.5	3.0 ± 0.9	−6.3 ± 2.55	+0.9 ± 0.2	−5.4 ± 2.66
	Group 2: Access flap + e‐PTFE membrane (*n* = 15)	0	8.2 ± 2.3	2.7 ± 1.0	3.6 ± 1.0	−5.5 ± 2.51	+1.0 ± 0.2	−4.6 ± 2.51
**Comparison of biomaterials used in regenerative surgery in the treatment of residual sites associated with infrabony defects**								
^15^	M‐MIST (*n* = 15)	0	7.5 ± 1.6	3.1 ± 0.6	3.0 ± 1.8	−4.4 ± 1.6	−0.1 ± 0.7	−4.5 ± 2.41
	b. Group 1: M‐MIST + EMD (*n* = 15)	0	7.8 ± 0.9	3.4 ± 0.6	3.4 ± 1.0	−4.4 ± 1.2	0.0 ± 0.9	−4.4 ± 1.35
	Group 2: M‐MIST + BMDX (*n* = 15)	1	7.3 ± 1.2	3.3 ± 0.6	3.2 ± 0.7	−4.0 ± 1.3	−0.1 ± 0.6	−4.1 ± 1.39
^17^	Regen with EMD + β‐TCP (*n* = 11)	Not reported	8.0 ± 1.2	3.5 ± 0.9	4.1 ± 0.9	−4.5 ± 1.5	0.6 ± 1.27	−3.9 ± 1.5
	b. Regen with EMD + NBM (*n* = 11)		8.1 ± 1.4	3.3 ± 0.7	4.1 ± 0.9	−4.8 ± 1.57	0.8 ± 1.14	−4.0 ± 1.66
^16^	Regen with autogenous BG alone (*n* = 20)	Not reported	7.3 ± 0.2	4.4 ± 0.3^†^	4.6 ± 0.5	−2.9 ± 0.4	0.2 ± 0.5^†^	−2.7 ± 0.5
	b. Regen with autogenous BG + GTR using a chair‐side‐prepared bioresorbable polylactic acid barrier extending 3 mm over the defect margins to cover the bone graft. (*n* = 20)^†^		7.6 ± 0.4	4.5 ± 0^†^	3.4 ± 0.3	−3.2 ± 0.4	−1.1 ± 0.5^†^	−4.2 ± 0.5
**Comparison of different surgical therapies in the treatment of residual sites not associated with infrabony defects** ^*^ ^,^ ^‡^ ^,^ ^§^								
^18^	MWF (*n* = 25)	Not reported	1‐4 mm: 3.21 ± 0.13 5‐6 mm: 5.35 ± 0.19 ≥7 mm: 7.17 ± 0.33	1‐4 mm: 1.90 ± 0.11 5‐6 mm: 2.80 ± 0.41 ≥7 mm: 3.85 ± 0.34^*^	1‐4 mm: 2.51 ± 0.61 5‐6 mm: 3.83 ± 0.55 ≥7 mm: 4.80 ± 0.45	1‐4 mm: ‐1.31 ± 0.17 5‐6 mm: ‐2.55 ± 0.45 ≥7 mm: ‐3.32 ± 0.47	1‐4 mm: +0.61 ± 0.62 5‐6 mm: +1.03 ± 0.69 ≥7 mm: 0.95 ± 0.56	1‐4 mm: ‐0.70 ± 0.62 5‐6 mm: ‐1.52 ± 0.58 ≥7 mm: ‐2.37 ± 0.56
	b. CAF + Adjunctive use of CO_2_ laser (*n* = 25)		1‐4 mm: 3.15 ± 0.21 5‐6 mm: 5.26 ± 0.22 ≥7 mm: 7.91 ± 0.81	1‐4 mm: 1.60 ± 0.43 5‐6 mm: 2.50 ± 0.53 ≥7 mm: 3.60 ± 0.51^*^	1‐4 mm: 1.95 ± 0.35 5‐6 mm: 2.80 ± 0.91 ≥7 mm: 4.00 ± 0.38	1‐4 mm: ‐1.55 ± 0.48 5‐6 mm: ‐2.76 ± 0.57 ≥7 mm: ‐4.31 ± 0.96	1‐4 mm: +0.35 ± 0.55 5‐6 mm: +0.30 ± 1.05 ≥7 mm: +0.40 ± 0.64	1‐4 mm: ‐1.20 ± 0.41 5‐6 mm: ‐2.46 ± 0.94 ≥7 mm: ‐3.91 ± 0.89
**Comparison of surgical and non‐surgical therapies in the treatment of residual sites not associated with infrabony defects**								
^19^	SRP (4‐6 sessions within 2 months using USS device) (*n* = 32)	Not reported	4.2 ± 1.0	3.1 ± 0.6	3.7 ± 0.6	−1.1 ± 1.17	+0.6 ± 0.85	0.5 ± 1.17
	b. MWF (4‐6 sessions within 2 months) (*n* = 32)		4.2 ± 0.8	2.6 ± 0.6	3.2 ± 0.9	−1.6 ± 1.0	+0.6 ± 1.08	1.0 ± 1.20

Reference	APT Control (*n* = no. of patients) Test (*n* = no. of patients)	CAL/ PAL baseline (mm)	CAL/ PAL at 1 year (^*^unless otherwise stated) (mm)	CAL/ PAL final (mm)	CAL/ PAL change from baseline to 1 year (^*^unless otherwise stated) (mm)	CAL/ PAL change from 1 year to final (^*^unless otherwise stated) (mm)	CAL/ PAL change from baseline to final SPC (mm)
**Comparison of regenerative surgery and OFD in the treatment of residual sites associated with infrabony defects**							
^10^	OFD (*n* = 15)	9.47 ± 2.27	6.00 ± 2.45	6.06 ± 1.85	+3.47 ± 2.80	−0.06 ± 2.44	+3.41 ± 2.75
	b. GTR using bioabsorbable polyactide acetyltributyl citrate barrier (*n* = 15)^*^	9.17 ± 1.51	5.50 ± 1.87	6.28 ± 1.24	+3.67 ± 2.11	−0.78 ± 1.93	+2.89 ± 2.12
^12^	OFD (*n* = 12)	9.41 ± 2.33	5.88 ± 2.47	5.88 ± 1.80	+3.53 ± 2.04	0.00 ± 2.83	+3.53 ± 2.50
	b. GTR using bioabsorbable polyactide acetyltributyl citrate barrier (*n* = 12)^*^	9.27 ± 1.58	5.20 ± 1.90	6.13 ± 2.27	+4.07 ± 2.88	−0.93 ± 0.66	+3.13 ± 2.22
^13^	OFD (*n* = 9)	10.4 ± 1.3	8.4 ± 1.2	8.6 ± 1.0	+2.0 ± 1.77	−0.2 ± 1.56	+1.8 ± 1.64
	b. Group 1: Regen using EMD (*n* = 10)	10.4 ± 1.6	7.0 ± 1.3	7.5 ± 1.4	+3.4 ± 2.06	−0.5 ± 1.91	+2.9 ± 2.13
	Group 2: Regen with GTR (using Revolut) (*n* = 10)	10.3 ± 1.6	7.1 ± 1.2	7.5 ± 1.2	+3.2 ± 2.00	−0.4 ± 1.70	+2.8 ± 2.0
	Group 3: Combination – EMD + GTR (*n* = 9)	10.2 ± 1.4	6.9 ± 1.1	7.3 ± 1.2	+3.3 ± 1.78	−0.4 ± 1.63	+2.9 ± 1.84
^14^	Access flap (MWF) (*n* = 15)	9.5 ± 2.7	7.1 ± 2.4	8.9 ± 3.2	+2.4 ± 3.61	−1.7 ± 0.4	+0.6 ± 4.18
	b. Group 1: Titanium‐reinforced e‐PTFE membranes + MPPT (*n* = 15)	9.9 ± 3.2	4.7 ± 1.8	4.9 ± 2.0	+5.2 ± 3.67	−0.1 ± 0.3	+5.0 ± 3.77
	Group 2: Access flap + e‐PTFE membrane (*n* = 15)	10.3 ± 2.4	6.3 ± 1.9	6.7 ± 2.1	+4.0 ± 3.06	−0.5 ± 0.1	+3.6 ± 3.19
**Comparison of biomaterials used in regenerative surgery in the treatment of residual sites associated with infrabony defects**							
^15^	M‐MIST (*n* = 15)	9.6 ± 2.0	5.5 ± 1.6	5.5 ± 2.0	+4.1 ± 1.4	−0.1 ± 0.7	+4.1 ± 2.83
	b. Group 1: M‐MIST + EMD (*n* = 15)	9.9 ± 1.3	5.7 ± 1.7	6.0 ± 1.4	+4.1 ± 1.2	−0.1 ± 0.8	+3.9 ± 1.91
	Group 2: M‐MIST + BMDX (*n* = 15)	10.1 ± 2.4	6.4 ± 2.4	6.5 ± 2.4	+3.7 ± 1.3	−0.3 ± 0.6	+3.6 ± 3.4
^17^	Regen with EMD + β‐TCP (*n* = 11)	8.9 ± 1.5	5.3 ± 0.9	5.8 ± 1.1	+3.6 ± 1.75	−0.5 ± 1.42	+3.1 ± 1.86
	b. Regen with EMD + NBM (*n* = 11)	9.1 ± 1.6	5.4 ± 1.1	6.1 ± 1.4	+3.7 ± 1.94	−0.7 ± 1.78	+3.0 ± 2.13
^16^	Regen with autogenous BG alone (*n* = 20)	9.2 ± 0.6	6.6 ± 0.6^¶^	7.0 ± 0.8	+2.5 ± 0.6	−0.4 ± 0.9^¶^	+2.2 ± 0.7
	b. Regen with autogenous BG + GTR using a chair‐side‐prepared bioresorbable polylactic acid barrier extending 3 mm over the defect margins to cover the bone graft. (*n* = 20)	9.0 ± 0.5	6.5 ± 0.6^¶^	5.2 ± 0.8	+2.5 ± 0.4	+1.2 ± 0.6^¶^	+3.8 ± 0.5
**Comparison of different surgical therapies in the treatment of residual sites not associated with infrabony defects** ^*^ ^,^ ^†^ ^,^ ^#^							
^18^	MWF (*n* = 25)	1‐4 mm: 3.18 ± 0.21 5‐6 mm: 6.11 ± 0.31 ≥7 mm: 8.63 ± 0.22	1‐4 mm: 3.49 ± 0.91 5‐6 mm: 5.50 ± 0.53 ≥7 mm: 7.29 ± 0.93^*^	1‐4 mm: 3.88 ± 0.23 5‐6 mm: 5.74 ± 0.21 ≥7 mm: 8.23 ± 0.63	1‐4 mm: ‐0.31 ± 0.93 5‐6 mm: +0.61 ± 0.61 ≥7 mm: +1.34 ± 0.96	1‐4 mm: ‐0.39 ± 0.94 5‐6 mm: ‐0.24 ± 0.57 ≥7 mm: ‐0.94 ± 1.12	1‐4 mm: ‐0.70 ± 0.31 5‐6 mm: +0.37 ± 0.37 ≥7 mm: +0.40 ± 0.67
	b. CAF + Adjunctive use of CO_2_ laser (*n* = 25)	1‐4 mm: 3.21 ± 0.16 5‐6 mm: 6.29 ± 0.43 ≥7 mm: 8.71 ± 0.34	1‐4 mm: 2.40 ± 0.76 5‐6 mm: 2.86 ± 1.06 ≥7 mm: 3.98 ± 1.12^*^	1‐4 mm: 2.78 ± 0.65 5‐6 mm: 2.55 ± 1.55 ≥7 mm: 3.61 ± 1.11	1‐4 mm: +0.81 ± 0.78 5‐6 mm: +3.43 ± 1.14 ≥7 mm: +4.73 ± 1.17	1‐4 mm: ‐0.38 ± 1.00 5‐6 mm: +0.31 ± 1.88 ≥7 mm: +0.37 ± 1.58	1‐4 mm: +0.43 ± 0.67 5‐6 mm: +3.74 ± 1.61 ≥7 mm: +5.10 ± 1.16
**Comparison of surgical and non‐surgical therapies in the treatment of residual sites not associated with infrabony defects**							
^19^	SRP (4‐6 sessions within 2 months using USS device) (*n* = 32)	Not reported					
	b. MWF (4‐6 sessions within 2 months) (*n* = 32)	Not reported					

Reference	APT Control (*n* = no. of patients) Test (*n* = no. of patients)	PROMs	FMPS (%)/ PI^∥^ baseline	FMPS (%)/ PI^∥^ final	FMBS (%)/ GI^∥^ baseline	FMBS (%)/ GI^∥^ final
**Comparison of regenerative surgery and OFD in the treatment of residual sites associated with infrabony defects**						
^10^	c. OFD (*n* = 15)	N/A	FMPS ≤ 25 0.82 ± 0.64	FMPS 44.79 1.06 ± 0.83	FMBS N/A 1.24 ± 0.66	FMBS 2.58 1.29 ± 0.59
	d. GTR using bioabsorbable polyactide acetyltributyl citrate barrier (*n* = 15)^*^ ^,^ ^**^ ^,^ ^††^	N/A	FMPS ≤ 25% 1.11 ± 0.83	FMPS (44.79) 0.83 ± 0.79	FMBS N/A 1.67 ± 0.59	FMBS 2.58 1.17 ± 0.38
^12^	c. OFD (*n* = 12)	N/A	FMPS ≤ 25 0.81 ± 0.66	FMPS (33.92) 0.94 ± 0.77	FMBS N/A 1.19 ± 0.66	FMBS 3.17 0.38 ± 0.50
	d. GTR using bioabsorbable polyactide acetyltributyl citrate barrier (*n* = 12))^*^	N/A	FMPS ≤ 25 1.00 ± 0.85	FMPS (33.92) 1.20 ± 0.68	FMBS N/A 1.67 ± 0.62	FMBS 3.17 0.37 ± 0.64
^13^	c. OFD (*n* = 9)	N/A N/A	0.6 ± 0.5^*^	1.0 ± 0.4^*^	1.7 ± 0.8^*^	1.0 ± 0.7^*^
	d. Group 1: Regen using EMD (*n* = 10)		0.8 ± 0.4^*^	1.2 ± 0.8^*^	1.7 ± 0.5^*^	1.0 ± 1.0^*^
	Group 2: Regen with GTR (using Revolut) (*n* = 10)		0.7 ± 0.5^*^	1.3 ± 0.7^*^	1.8 ± 0.8^*^	1.1 ± 0.9^*^
	Group 3: Combination – EMD + GTR (*n* = 9)		0.9 ± 0.3^*^	1.1 ± 0.8^*^	1.6 ± 0.5^*^	1.0 ± 0.8^*^
^14^	c. Access flap (MWF) (*n* = 15)	N/A	12.5 ± 3.6	9.2 ± 3.1	8.7 ± 3.2	7.2 ± 3
	d. Group 1: Titanium‐reinforced e‐PTFE membranes + MPPT (*n* = 15)	N/A	12.2 ± 1.2	9.6 ± 2.7	10.2 ± 2	7.1 ± 2.2
	Group 2: Access flap + e‐PTFE membrane (*n* = 15)	N/A	11 ± 2.3	9.2 ± 3.1	10.9 ± 3.2	7.2 ± 3
**Comparison of biomaterials used in regenerative surgery in the treatment of residual sites associated with infrabony defects**						
^15^	c. M‐MIST (*n* = 15)	N/A	13.6 ± 4.9	8.7 ± 2.1	10.3 ± 4.4	6.9 ± 2.1
	d. Group 1: M‐MIST + EMD (*n* = 15)	N/A	12.5 ± 3.7	9.3 ± 2.7	10.4 ± 3.4	7.2 ± 2.9
	Group 2: M‐MIST + BMDX (*n* = 15)	N/A	14.4 ± 6.0	9.7 ± 2.8	10.7 ± 4.1	6.7 ± 2.7
^17^	c. Regen with EMD + β‐TCP (*n* = 11)	N/A N/A	0.4 ± 0.3^*^	0.9 ± 0.5^*^	1.0 ± 0.3^*^	0.8 ± 0.5^*^
	d. Regen with EMD + NBM (*n* = 11)		0.5 ± 0.2^*^	0.8 ± 0.4^*^	1.1 ± 0.2^*^	0.7 ± 0.9^*^
^16^	c. Regen with autogenous BG alone (*n* = 20)	N/A	0.5 ± 0.1^†^	0.4 ± 0.1^†^	0.9 ± 0.1^†^	0.5 ± 0.1^†^
	d. Regen with autogenous BG + GTR using a chair‐side‐prepared bioresorbable polylactic acid barrier extending 3 mm over the defect margins to cover the bone graft. (*n* = 20)		0.5 ± 0.1^†^	0.5 ± 0.1^†^	0.9 ± 0.2^†^	0.9 ± 0.1^†^
**Comparison of different surgical therapies in the treatment of residual sites not associated with infrabony defects**						
^18^	c. MWF (*n* = 25)	N/A N/A	1.02 ± 0.43^*^ ^,^ ^‡^ ^,^ ^§^	1.26 ± 0.49^*^ ^,^ ^‡^ ^,^ ^§^	1.86 ± 0.62^*^ ^,^ ^‡^ ^,^ ^§^	1.07 ± 0.62^*^ ^,^ ^‡^ ^,^ ^§^
	d. CAF + Adjunctive use of CO_2_ laser (*n* = 25)		1.02 ± 0.43^*^ ^,^ ^‡^ ^,^ ^§^	1.33 ± 0.76^*^ ^,^ ^‡^ ^,^ ^§^	1.86 ± 0.62^*^ ^,^ ^‡^ ^,^ ^§^	1.10 ± 0.54^*^ ^,^ ^‡^ ^,^ ^§^
**Comparison of surgical and non‐surgical therapies in the treatment of residual sites not associated with infrabony defects**						
^19^	c. SRP (4‐6 sessions within 2 months using USS device) (*n* = 32)	N/A				
	d. MWF (4‐6 sessions within 2 months) (*n* = 32)	N/A				

Abbreviations: BG, bone graft; BMDX, bone mineral derived xenograft; CAF, coronally advanced flap; EMD, enamel matrix derivative; GTR, guided tissue regeneration; M‐MIST, modified minimally invasive surgical therapy; MPPT, modified papilla preservation technique; MWF, modified Widman flap; NBM, natural bone mineral; NSPT, non‐surgical periodontal therapy; OFD, open flap debridement; Regen, regenerative surgery; SRP, scaling and root planing; USS, ultrasonic; β‐TCP, β‐tricalcium phosphate.

^*^ Split‐mouth design.

^†^ PPD at 9 months.

^‡^ Stratified into baseline PPD 1–4 mm, 5–6 mm, ≥7 mm.

^§^ PPD at 6 months.

^¶^ CAL at 9 months.

^#^ CAL at 6 months.

^∥^ Not reported in the study but calculated using means ± SD of the baseline, 1‐year (or equivalent), and final SPC visits.

^**^ PI only treated teeth.

†† SBI only treated teeth.

#### 2.5.2 Risk of bias assessment

Quality assessment was conducted by 1 reviewer (J.L.) and independently reviewed by V.R. The included studies were evaluated using the Cochrane Risk of Bias (RoB) 2 Tool for randomized controlled trials.^20^ Any disagreements were resolved through consensus, with an arbitrator (L.N.) consulted if consensus could not be reached.

#### 2.5.3 Data analyses

A descriptive analysis of all data was performed. In case of a minimum of 3 similar studies, a meta‐analysis of weighted mean difference (WMD) was undertaken. Meta‐analyses were performed using IBS SPSS 29.0, to determine WMD in relation to tooth loss (if mean and SD of tooth loss was provided), PPD and CAL (of raw data at baseline and endpoint of treatment) between treatment and control groups at the endpoint of the study. In cases where studies included multiple test groups, the test group most comparable to those in other studies was selected for analysis. When publications of the same trial were considered, the article with stricter compliance with SPC were included in the analyses to avoid duplicating data. Subgroup analyses of the different follow‐up intervals, which were conducted to investigate potential sources of heterogeneity, included all trials. WMDs and the corresponding 95% confidence intervals (CIs) were calculated as effect sizes. With treatment outcome affected by subject‐, tooth‐ and treatment‐related factors, a random‐effects model was deemed appropriate to calculate the average distribution of mean effects, based on clinical and statistical reasoning.^21^

The extent and impact of inter‐study heterogeneity were evaluated by examining forest plots and calculating τ^2^ (absolute heterogeneity) and I^2^ (relative heterogeneity). I^2^ values exceeding 75% were arbitrarily considered indicative of substantial heterogeneity.^22^

Where both parallel‐group and split‐mouth RCTs were included in the meta‐analyses, the unit of analysis was the tooth/ defect rather than the patient. This allows for valid comparison with parallel‐group studies without risk of clustering bias. As such, all included studies were treated as having independent observations at the site‐level.

## 3 RESULTS

### 3.1 Study selection

**FIGURE 1:**
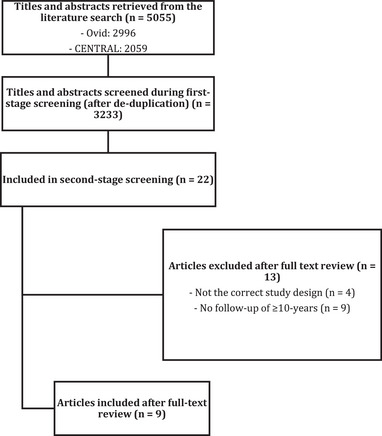
Flowchart detailing screening process.

The initial search yielded 5055 articles from all sources combined (Figure 1). Following the initial screening, 22 articles qualified for full‐text review and 9 articles for subsequent inclusion.^10^, ^12^, ^13^, ^14^, ^15^, ^16^, ^17^, ^18^, ^19^ The Cohen's kappa value for inter‐reviewer agreement was 0.95 at first‐stage screening and 0.91 at second‐stage screening.

The publication year ranged from 2001 to 2022. All 9 papers were conducted in Europe, and 2 were based on the same RCT with a 10‐year follow‐up period^10^ and 20‐year follow‐up period.^12^ Demographics typically included a mix of male and female participants, with most participants being systemically healthy.

RCTs with split‐mouth designs were undertaken in 3 studies.^10^, ^12^, ^18^ Four studies were based in private practice^14^, ^15^, ^16^, ^18^ and 5 studies in university hospital.^10^, ^12^, ^13^, ^17^, ^19^

Four RCTs focused on comparing regenerative surgery, utilizing GTR in at least 1 of the test arms, and OFD in the treatment of residual sites associated with intra‐bony defects.^10^, ^12^, ^13^, ^14^ Three RCTs analyzed different biomaterials in the regenerative treatment of residual sites associated with intra‐bony defects.^15^, ^16^, ^17^ One study focused on comparing a surgical technique (modified Widman flap [MWF]) against scaling and root planing (SRP) of non‐molar teeth^19^ and another compared 2 surgical techniques (MWF vs. coronally advanced flap (CAF) and CO_2_ laser irradiation of the roots) in the treatment of teeth with PPD ≥5 mm.^18^

SPC frequency varied from 3‐ to 6‐monthly in the mid‐ to long‐term for most studies. Two studies reported SPC for 3‐monthly in the first year and then approximately once a year with the patients’ GDPs thereafter (Nickles et al., 2009; Petsos et al., 2019). These sessions generally consisted of oral hygiene reinforcement, supragingival ± subgingival professional mechanical plaque removal (PMPR) under local anesthetic and, when required, occlusal adjustment and fluoride application.

### 3.2 Tooth loss

Four studies reported on tooth loss, 3 of which compared regenerative procedures with an access flap approach.^10^, ^12^, ^14^ The fourth study examined different regenerative techniques, focusing on groups that employed the same flap approach (M‐MIST) but used various or no biomaterials.^15^ Across all studies, a reported range of 0–4 teeth were lost across all 4 studies in test and control groups. A meta‐analysis comparing WMD of tooth loss was not performed as only 2 independent studies were available for analysis, and there was a lack of reported means and standard deviations. The remaining 5 studies did not report on tooth loss as an outcome measure, which presents a limitation in evaluating long‐term effectiveness of the periodontal interventions examined.^13^, ^16^, ^17^, ^18^, ^19^

### 3.3 PPD reduction

Of the 9 studies included, all reported PPD measurements at baseline, 1 year and after the final SPC follow‐up, except for 2 studies.^16^, ^18^ Instead of 1‐year follow‐ups, these 2 studies reported clinical measurements at 6‐month^18^ and 9‐month^16^ follow‐ups.

When comparing regenerative approaches to OFD across 4 studies, test and control groups demonstrated varied but significant PPD reductions at 1 year. Over an extended follow‐up period from 1 to 20 years, there was a marked variability in PPD stability across groups. Test groups employing GTR with various flaps showed a modest increase in PPD of 0.9 ± 0.2 mm to 1.0 ± 0.2 mm, while the OFD group experienced a larger increase of 1.9 ± 0.6 mm between 1‐ and 20‐year follow‐ups.^14^ In contrast, a study with 10‐ and 20‐year follow‐ups demonstrated different results.^12^ The 10‐year counterpart found continuous and greater PPD reductions in the OFD group (−0.71 ± 2.49 mm) at 10 years during SPC relative to the regenerative group (−0.11 ± 1.91 mm).^10^ When comparing combinations of enamel matrix derivative (EMD) ± GTR to OFD, increases in PPD from 1‐year to 10‐year follow‐ups was illustrated across groups, with the OFD group experiencing the smallest PPD increase of 0.2 ± 2.16 mm amongst study arms. Therefore, similar PPD reductions were evident between baseline and final visit varying between ‐3.4 ± 1.97 mm and ‐3.6 m ± 2.2 mm between all 4 treatment arms.^13^

**FIGURE 2:**
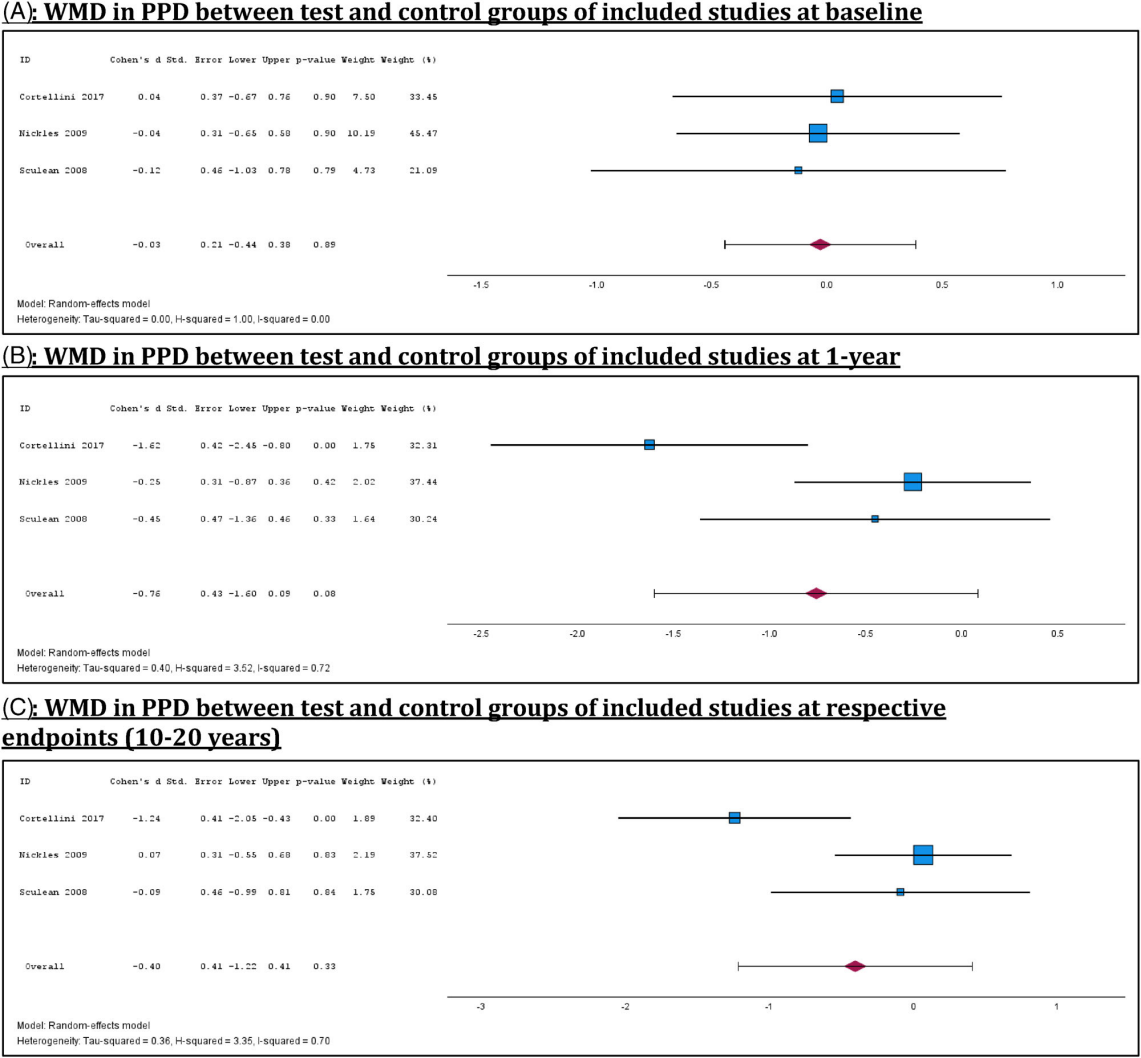
Meta‐analyses of WMD in PPD between test and control groups at baseline, 1‐year and respective endpoints. (A) WMD in PPD between test and control groups of included studies at baseline. (B) WMD in PPD between test and control groups of included studies at 1 year. (C) WMD in PPD between test and control groups of included studies at respective endpoints (10–20 years).

Meta‐analyses of 3 studies comparing GTR and OFD found no statistically significant difference in PPD between the test and control groups at baseline, confirming the comparability of treatment and control arms across studies (Figure 2A).^10^, ^13^, ^14^ At both the 1‐year mark and long‐term follow‐ups of 10–20 years, there were similarly no statistically significant differences in PPD between the test and control groups, indicating that both treatment approaches yielded comparable clinical outcomes over extended periods (Figure  2B,C). However, substantial heterogeneity was observed in both meta‐analyses (*I* ^2^ = 0.70 – 0.72). Sub‐analyses of trials which compared OFD and GTR at 10 years demonstrated no statistically significant differences in PPD reductions at both the 1‐year mark and respective endpoints of trials (Supplemental Material 3 in the online *Journal of Periodontology*).

All 3 studies that compared various or a lack of biomaterials in the treatment of residual sites associated with infra‐bony defects reported substantial PPD reductions across treatment groups at the initial post‐treatment follow‐up at 1 year (or equivalent) compared to baseline values. Whereas the magnitude of PPD reductions differed between studies, the results within each individual study were relatively consistent between the test and control arms, indicating that the effectiveness of each intervention was comparable within the context of the respective study's treatment groups. However, stability of the PPD reduction varied. Whilst Cortellini and colleagues were able to demonstrate stable reductions across all test and control arms at 10‐year follow‐up,^15^ this trend was not consistently observed in other studies. Döri's group demonstrated an increase in PPD ranging from 0.6 ± 1.27 mm in the control group to 0.8 ± 1.14 mm in the test group between the 1‐year and 10‐year follow‐ups.^17^ In contrast, others demonstrated comparable PPD reductions at 9 months, but their test group (autogenous bone graft + GTR) maintained a minor additional reduction of −1.1 mm ± 0.5 mm.^16^ Thus, despite variations in absolute PPD reductions across studies, each study demonstrated a similar pattern of PPD reduction within its respective treatment and control groups which were generally maintained at 10‐year follow‐ups. The consistency within studies highlights that while different regenerative approaches may vary in their absolute effects, they can each offer significant and comparable improvements when supported by strict SPC.

A 15‐year follow‐up of patients treated with either MWF or a CAF combined with CO_2_ laser root irradiation, a split‐mouth design was used and assessed the effectiveness of each technique for sites with moderate to advanced periodontitis using PPD thresholds of PPD 1–4 mm, PPD 5–6 mm, and PPD ≥7 mm. Both surgical modalities demonstrated similar PPD reductions. Yet, the CAF + CO_2_ laser adjunct test group demonstrated slightly less relapse in PPD measurements in PPD ≥5 mm between 6‐month and 15‐year follow‐ups.^18^ In a 13‐year follow‐up study comparing SRP with MWF, similar PPD increases across groups were seen between 1 and 13 years of 0.6 ± 0.85 mm and 0.6 ± 1.08 mm, respectively.^19^

### 3.4 CAL gain

Eight studies included full‐mouth CAL data between baseline, 1‐year (or equivalent) and final follow‐ups.^10^, ^12^, ^13^, ^14^, ^15^, ^16^, ^17^, ^18^

**FIGURE 3:**
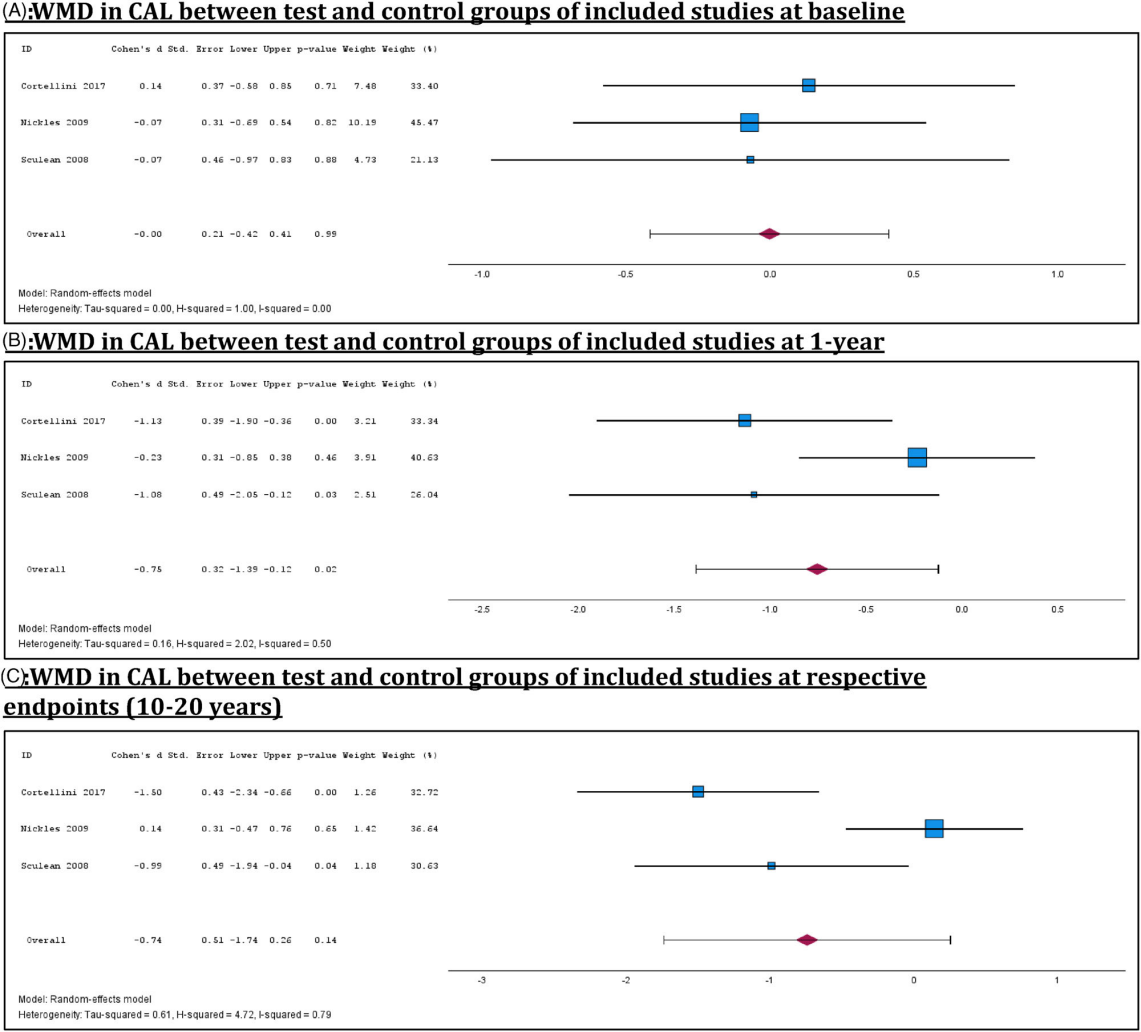
Meta‐analyses of WMD in CAL between test and control groups at baseline, 1 year, and respective endpoints. (A) WMD in CAL between test and control groups of included studies at baseline. (B) WMD in CAL between test and control groups of included studies at 1 year. (C) WMD in CAL between test and control groups of included studies at respective endpoints (10–20 years).

Meta‐analyses of 3 studies comparing GTR and OFD found no statistically significant difference in CAL between the test and control groups at baseline, confirming the equivalence of treatment and control arms across studies (Figure 3a).^10^, ^13^, ^23^ At 1‐year follow‐up, the pooled WMD showed a modest and‐statistically significant (WMD: −0.75; *p *= 0.02) improvement in CAL gain favoring the GTR groups (Figure 3b). Yet long‐term follow‐up of 10–20 years revealed no statistically significant difference in CAL gain between the interventions (WMD: −0.74; *p* = 0.14), suggesting both treatment modalities achieved similar long‐term outcomes. However, significant heterogeneity between studies (I^2^ = 0.79) was observed (Figure 3c). Sub‐analyses of trials comparing OFD and GTR at 10‐year SPC follow‐up demonstrated no statistically significant differences in CAL gains at both the 1‐year mark and respective endpoints of trials (Supplemental Material 4 in the online *Journal of Periodontology*).

Across all studies analyzing various regenerative materials used in the management of infra‐bony defects, similar CAL gains were achieved between test and control arms of individual studies. CAL stability between the 1‐year (or equivalent) and final follow‐up periods demonstrated minimal CAL loss between 0.1 ± 0.7 mm and 0.7 ± 1.78 mm in regenerative procedures without the use of GTR.^15^, ^17^ The GTR test‐arm was the only group across all 3 studies demonstrating a continued gain in CAL of 1.2 ± 0.6 mm between 9‐month and 10‐year follow‐ups.^16^

CAL gains when comparing MWF and CAF + CO_2_ laser root irradiation demonstrated more modest CAL improvements in deeper sites (PPD ≥7 mm) of 1.34 ± 0.96 mm at 6 months in the control group (MWF) versus 3.98 ± 1.12 mm CAL gain in the test group (CAF + CO_2_ laser root irradiation). Continued and sustained CAL gains of 0.37 ± 1.58 mm were evident between 6 months and 15 years in sites with PPD ≥7 mm in the test group, outperforming the MWF control in terms of CAL improvement and long‐term stability.^18^

### 3.5 PROMs

None of the included studies reported on PROMs.

### 3.6 Risk of bias assessment

Supplemental Material 5 in the online *Journal of Periodontology* reports risk of bias assessments for RCTs. RCTs demonstrated high risk of bias in most studies, mainly as a consequence of missing data at the final follow‐up. Based on the GRADE approach (Supplemental Material 6 in the online *Journal of Periodontology*), the overall certainty of evidence varied across outcomes. Tooth loss data were rated as having very low certainty due to high risk of bias, inconsistency, and imprecision. PPD reduction and CAL gain at 10–20 years of follow‐up were graded as low certainty due to concerns about study heterogeneity.

## 4 DISCUSSION

This systematic review investigated whether periodontal interventions applied during APT provide long‐term clinical benefit beyond those achieved with the standard control intervention in patients undergoing long‐term SPC within RCTs. Given the chronic and recurrent nature of periodontitis, understanding the sustained effectiveness of APT modalities in the context of SPC is essential. Our findings offer insight into how different therapeutic strategies perform over extended follow‐up periods when embedded within rigorous maintenance protocols.

OFD and resective surgical interventions, in the short‐term, have often demonstrated superior reductions in PPD and CAL compared to control treatments.^24^, ^25^ However, this initial benefit was frequently attenuated over the medium‐term, with comparable outcomes between test and control arms.^5^, ^6^, ^7^ Current guidelines recommend regenerative techniques for patients with deep intra‐bony defects to achieve superior attachment gain and pocket depth reduction after a minimum follow‐up of 12 months.^26^ Regenerative surgical techniques, by a single research group, tend to show long‐term benefits.^14^, ^15^, ^27^ However, meta‐analyses comparing GTR and OFD, within this systematic review (Figures 2C and 3C), found no significant difference in PPD or CAL at long‐term follow‐ups, challenging the assumption that regenerative procedures confer superior enduring outcomes. These findings suggest that while different surgical interventions may offer varied short‐term benefits, their long‐term effectiveness appears to converge when embedded within a structured SPC program. This underscores the potential role of high‐quality, sustained maintenance care in preserving periodontal health, potentially outweighing the differences between surgical modalities over time. However, as all included studies incorporated some form of SPC, and no comparison group without maintenance was available, this interpretation remains indirect. Therefore, while the results underscore the association between consistent SPC and stable long‐term outcomes, causality cannot be inferred, and the role of SPC should be interpreted cautiously within the context of the available data.

Notably, SPC intervals within the studies included in these meta‐analyses varied among groups, ranging from more than once a year after the first year^10^ to 3‐monthly intervals.^13^, ^14^ It is plausible that the absence or inconsistency of SPC contributed to the relapse in PPD reductions observed from the 1‐year mark to the end of the trials. Yet, interestingly, the OFD group continued to show PPD improvements, suggesting that continued improvements following regenerative techniques may be more heavily dependent on strict long‐term SPC. Nevertheless, 2 studies with consistent 3‐monthly SPC intervals throughout the trial period reported contrasting findings regarding the magnitude of PPD relapse between the control and test groups from the 1‐year mark to the trial endpoint.^13^, ^14^ These findings suggest that while regenerative techniques may initially provide superior PPD outcomes, their long‐term success is likely governed by the quality and consistency of SPC and patient‐related risk factors such as compliance and risk profile. This underscores the multifactorial nature of periodontal stability.

Individual studies comparing various regenerative biomaterials, such as EMD, autogenous bone and bioresorbable membranes, also showed early improvements across test arms. Yet only a subset demonstrated sustained benefits, suggesting that the use of biomaterials, alone, may not ensure long‐term success unless embedded within an effective maintenance regimen. Notably, in a single study, GTR with a bioresorbable membrane and autogenous bone led to a continued CAL gain over 10 years, whereas the control arm stabilized without further improvement.^16^ This highlights the potential for tailored regenerative strategies when combined with consistent SPC.

The results demonstrated that both surgical and non‐surgical therapies can effectively maintain long‐term periodontal stability in noninfra‐bony defects when supported by rigorous SPC.^18^, ^19^ While the CAF + CO_2_ laser approach showed slightly superior long‐term PPD stability compared to MWF, others found no significant difference between MWF and SRP over 13 years. These findings suggest that non‐surgical therapy may be sufficient for managing noninfra‐bony defects in patients who are committed to SPC, with surgical options potentially reserved for cases where additional stability is required or where adjunctive therapies, like CO_2_ laser, can offer additional benefit.

Despite the inclusion of long‐term studies, reporting of tooth loss outcomes was inconsistent, limiting the capacity to evaluate hard endpoints across interventions. Nevertheless, the minimal number of teeth lost in studies that did report on this outcome suggest that treatments, when supported by consistent SPC, are effective in preserving the dentition over time. This has also been confirmed elsewhere.^28^, ^29^

This review highlights the interplay between initial treatment modality and SPC as a determinant of long‐term success in periodontal therapy. The limited added benefit of regenerative approaches beyond the first year, as shown in both clinical and meta‐analytical comparisons, raises questions about their routine use, especially considering their higher costs and technical demands. These findings may encourage a more selective and personalized application of regenerative therapies, targeting patients likely to derive long‐term benefit based on compliance, risk profile, and defect characteristics.

Limitations, outside of the limited number of studies included, are the substantial heterogeneity across studies in terms of defect types, surgical protocols including the use of various grafting materials when regenerative surgeries were employed, and SPC intervals. These impair the generalizability of pooled estimates. Methodological issues, inconsistency, and imprecision in the included studies which resulted in GRADE ratings of very low to moderate further tempers the strength of conclusions. Moreover, none of the included RCTs reported PROMs, an important omission given the shift toward patient‐centered care.

To advance the evidence base, future research should prioritize well‐designed RCTs with standardized SPC protocols and long‐term follow‐up durations. Trials should stratify by patient compliance, operator experience, and biomaterial type to clarify which combinations yield the most durable outcomes. Future trials should integrate PROMs alongside clinical endpoints to better capture the full impact of periodontal therapies. Furthermore, economic evaluations comparing the cost‐effectiveness of regenerative versus non‐regenerative approaches in the context of SPC are warranted to inform resource allocation and clinical decision‐making in real‐world settings.

## 5 CONCLUSION

This review emphasizes that periodontal interventions during APT, whether regenerative or non‐regenerative, surgical or non‐surgical, can yield lasting clinical benefits when supported by stringent SPC. Whereas test interventions offered short‐term advantages, their long‐term benefits appeared, more often than not, comparable to standard control interventions when supported by SPC. These findings highlight the potential importance of SPC in maintaining periodontal stability over time. However, given the small number of included studies and the low to very low certainty of evidence, these conclusions should be interpreted with caution. Further high‐quality, long‐term RCTs are needed to confirm these observations and to better inform clinical decision‐making regarding the added value of complex or cost‐intensive interventions.

## AUTHOR CONTRIBUTIONS

Varkha Rattu and Luigi Nibali conceived this systematic review and co‐designed the review protocol. Varkha Rattu created the search strategy. Tishani Patel, Jasmine Loke, and Varkha Rattu performed the literature search. Jasmine Loke performed the risk of bias assessments. Varkha Rattu extracted the data and prepared the draft manuscript, which was reviewed and edited by Tishani Patel, Jasmine Loke, Hari Petsos, and Luigi Nibali. Luigi Nibali supervised, reviewed, and provided commentary or revisions at each stage.

## CONFLICT OF INTEREST STATEMENT

The authors declare no conflicts of interest. No external funding was received for this study.

## FUNDING INFORMATION

The authors received no specific funding for this work.

## Supporting information



Supporting Information

## Data Availability

The data that support the findings of this study are available in the tables, figures, and supplemental material of this article.
