# Supplementary material for: Long‐term clinical benefits of periodontal interventions in strict supportive periodontal care: A systematic review
Source: J Periodontol. 2025 Nov 18;97(3):450–72. doi: 10.1002/jper.70027 (PMC13111774; doi:10.1002/jper.70027)
Supplement: Supplementary file 1 — Supporting Information [file JPER-97-450-s001.docx]

**Supplemental Material 1: Search strategies**

**S1.1: Search Strategy in Ovid EMBASE (05.12.2023)**

| \| 1. \| exp randomized controlled trial/ \| \| --- \| --- \| \| 2. \| Controlled clinical trial/ \| \| 3. \| random$.ti,ab. \| \| 4. \| randomization/ \| \| 5. \| intermethod comparison/ \| \| 6. \| placebo.ti,ab. \| \| 7. \| (compare or compared or comparison).ti. \| \| 8. \| ((evaluated or evaluate or evaluating or assessed or assess) and (compare or compared or comparing or comparison)).ab. \| \| 9. \| (open adj label).ti,ab. \| \| 10. \| ((double or single or doubly or singly) adj (blind or blinded or blindly)).ti,ab. \| \| 11. \| double blind procedure/ \| \| 12. \| parallel group$1.ti,ab. \| \| 13. \| (crossover or cross over).ti,ab. \| \| 14. \| ((assign$ or match or matched or allocation) adj5 (alternate or group$1 or intervention$1 or patient$1 or subject$1 or participant$1)).ti,ab. \| \| 15. \| (assigned or allocated).ti,ab. \| \| 16. \| (controlled adj7 (study or design or trial)).ti,ab. \| \| 17. \| (volunteer or volunteers).ti,ab. \| \| 18. \| human experiment/ \| \| 19. \| trial.ti. \| \| 20. \| or/1-19 \| \| 21. \| (random$ adj sampl$ adj7 ("cross section$" or questionnaire$1 or survey$ or database$1)).ti,ab. not (comparative study/ or controlled study/ or randomi?ed controlled.ti,ab. or randomly assigned.ti,ab.) \| \| 22. \| Cross-sectional study/ not (exp randomized controlled trial/ or controlled clinical study/ or controlled study/ or randomi?ed controlled.ti,ab. or control group$1.ti,ab.) \| \| 23. \| (((case adj control$) and random$) not randomi?ed controlled).ti,ab. \| \| 24. \| Systematic review.ti,ab. not (trial or study).ti. \| \| 25. \| (nonrandom$ not random$).ti,ab. \| \| 26. \| "random field$".ti,ab. \| \| 27. \| (random cluster adj3 sampl$).ti,ab. \| \| 28. \| (review.ab. and review.pt.) not trial.ti. \| \| 29. \| "we searched".ab. and (review.ti. or review.pt.) \| \| 30. \| "update review".ab. \| \| 31. \| (databases adj4 searched).ab. \| \| 32. \| (rat or rats or mouse or mice or swine or porcine or murine or sheep or lambs or pigs or piglets or rabbit or rabbits or cat or cats or dog or dogs or cattle or bovine or monkey or monkeys or trout or marmoset$1).ti. and animal experiment/ \| \| 33. \| Animal experiment/ not (human experiment/ or human/) \| \| 34. \| or/21-33 \| \| 35. \| 20 not 34 \| \| 36. \| exp periodontal disease/ \| \| 37. \| exp periodontitis/ \| \| 38. \| exp alveolar bone loss/ \| \| 39. \| ((periodontal adj disease*) or periodontitis).mp. [mp=title, abstract, heading word, drug trade name, original title, device manufacturer, drug manufacturer, device trade name, keyword heading word, floating subheading word, candidate term word] \| \| 40. \| (periodont* adj2 pocket*).mp. [mp=title, abstract, heading word, drug trade name, original title, device manufacturer, drug manufacturer, device trade name, keyword heading word, floating subheading word, candidate term word] \| \| 41. \| (alveolar adj2 loss).mp. [mp=title, abstract, heading word, drug trade name, original title, device manufacturer, drug manufacturer, device trade name, keyword heading word, floating subheading word, candidate term word] \| \| 42. \| (alveolar adj2 atroph*).mp. [mp=title, abstract, heading word, drug trade name, original title, device manufacturer, drug manufacturer, device trade name, keyword heading word, floating subheading word, candidate term word] \| \| 43. \| (attachment adj3 loss).mp. [mp=title, abstract, heading word, drug trade name, original title, device manufacturer, drug manufacturer, device trade name, keyword heading word, floating subheading word, candidate term word] \| \| 44. \| exp dental scaling/ \| \| 45. \| exp curettage/ \| \| 46. \| exp dental curettage/ \| \| 47. \| exp debridement/ \| \| 48. \| exp dental debridement/ \| \| 49. \| exp mechanical debridement/ \| \| 50. \| ((non surgical adj4 therap*) or (nonsurgical adj4 therap*)).mp. [mp=title, abstract, heading word, drug trade name, original title, device manufacturer, drug manufacturer, device trade name, keyword heading word, floating subheading word, candidate term word] \| \| 51. \| ((non surgical adj4 treatment*) or (nonsurgical adj4 treatment*)).mp. [mp=title, abstract, heading word, drug trade name, original title, device manufacturer, drug manufacturer, device trade name, keyword heading word, floating subheading word, candidate term word] \| \| 52. \| ((non surgical adj4 care) or (nonsurgical adj4 care)).mp. [mp=title, abstract, heading word, drug trade name, original title, device manufacturer, drug manufacturer, device trade name, keyword heading word, floating subheading word, candidate term word] \| \| 53. \| ((non surgical adj4 maintenance) or (nonsurgical adj4 maintenance)).mp. [mp=title, abstract, heading word, drug trade name, original title, device manufacturer, drug manufacturer, device trade name, keyword heading word, floating subheading word, candidate term word] \| \| 54. \| (non surgical periodontal therap* or nonsurgical periodontal therap*).mp. [mp=title, abstract, heading word, drug trade name, original title, device manufacturer, drug manufacturer, device trade name, keyword heading word, floating subheading word, candidate term word] \| \| 55. \| NSPT.mp. \| \| 56. \| ((supragingival adj3 scal*) or (supra gingival adj3 scal*)).mp. [mp=title, abstract, heading word, drug trade name, original title, device manufacturer, drug manufacturer, device trade name, keyword heading word, floating subheading word, candidate term word] \| \| 57. \| ((supragingival adj3 debrid*) or (supra gingival adj3 debrid*)).mp. [mp=title, abstract, heading word, drug trade name, original title, device manufacturer, drug manufacturer, device trade name, keyword heading word, floating subheading word, candidate term word] \| \| 58. \| ((supragingival adj3 curett*) or (supra gingival adj3 curett*)).mp. [mp=title, abstract, heading word, drug trade name, original title, device manufacturer, drug manufacturer, device trade name, keyword heading word, floating subheading word, candidate term word] \| \| 59. \| (subgingival adj3 scal*).mp. [mp=title, abstract, heading word, drug trade name, original title, device manufacturer, drug manufacturer, device trade name, keyword heading word, floating subheading word, candidate term word] \| \| 60. \| (subgingival adj3 debrid*).mp. [mp=title, abstract, heading word, drug trade name, original title, device manufacturer, drug manufacturer, device trade name, keyword heading word, floating subheading word, candidate term word] \| \| 61. \| (subgingival adj3 curett*).mp. [mp=title, abstract, heading word, drug trade name, original title, device manufacturer, drug manufacturer, device trade name, keyword heading word, floating subheading word, candidate term word] \| \| 62. \| (root adj2 planing).mp. [mp=title, abstract, heading word, drug trade name, original title, device manufacturer, drug manufacturer, device trade name, keyword heading word, floating subheading word, candidate term word] \| \| 63. \| professional mechanical plaque removal.mp. \| \| 64. \| PMPR.mp. \| \| 65. \| (professional adj2 plaque removal).mp. [mp=title, abstract, heading word, drug trade name, original title, device manufacturer, drug manufacturer, device trade name, keyword heading word, floating subheading word, candidate term word] \| \| 66. \| (periodont* adj3 therap*).mp. [mp=title, abstract, heading word, drug trade name, original title, device manufacturer, drug manufacturer, device trade name, keyword heading word, floating subheading word, candidate term word] \| \| 67. \| (periodont* adj3 treatment*).mp. [mp=title, abstract, heading word, drug trade name, original title, device manufacturer, drug manufacturer, device trade name, keyword heading word, floating subheading word, candidate term word] \| \| 68. \| (periodont* adj3 care).mp. [mp=title, abstract, heading word, drug trade name, original title, device manufacturer, drug manufacturer, device trade name, keyword heading word, floating subheading word, candidate term word] \| \| 69. \| exp maintenance therapy/ \| \| 70. \| supportive periodontal care.mp. \| \| 71. \| SPC.mp. \| \| 72. \| supportive periodontal treatment*.mp. \| \| 73. \| supportive periodontal therap*.mp. \| \| 74. \| SPT.mp. \| \| 75. \| (maintenance adj3 therap*).mp. [mp=title, abstract, heading word, drug trade name, original title, device manufacturer, drug manufacturer, device trade name, keyword heading word, floating subheading word, candidate term word] \| \| 76. \| (maintenance adj3 treatment*).mp. [mp=title, abstract, heading word, drug trade name, original title, device manufacturer, drug manufacturer, device trade name, keyword heading word, floating subheading word, candidate term word] \| \| 77. \| (maintenance adj3 care).mp. [mp=title, abstract, heading word, drug trade name, original title, device manufacturer, drug manufacturer, device trade name, keyword heading word, floating subheading word, candidate term word] \| \| 78. \| (maintenance adj3 recall*).mp. [mp=title, abstract, heading word, drug trade name, original title, device manufacturer, drug manufacturer, device trade name, keyword heading word, floating subheading word, candidate term word] \| \| 79. \| exp follow up/ \| \| 80. \| follow up.mp. \| \| 81. \| followup.mp. \| \| 82. \| 36 or 37 or 38 or 39 or 40 or 41 or 42 or 43 \| \| 83. \| 44 or 45 or 46 or 47 or 48 or 49 or 50 or 51 or 52 or 53 or 54 or 55 or 56 or 57 or 58 or 59 or 60 or 61 or 62 or 63 or 64 or 65 or 66 or 67 or 68 \| \| 84. \| 69 or 70 or 71 or 72 or 73 or 74 or 75 or 76 or 77 or 78 or 79 or 80 or 81 \| \| 85. \| 35 and 82 and 83 and 84 \| |
| --- | --- | --- | --- | --- | --- | --- | --- | --- | --- | --- | --- | --- | --- | --- | --- | --- | --- | --- | --- | --- | --- | --- | --- | --- | --- | --- | --- | --- | --- | --- | --- | --- | --- | --- | --- | --- | --- | --- | --- | --- | --- | --- | --- | --- | --- | --- | --- | --- | --- | --- | --- | --- | --- | --- | --- | --- | --- | --- | --- | --- | --- | --- | --- | --- | --- | --- | --- | --- | --- | --- | --- | --- | --- | --- | --- | --- | --- | --- | --- | --- | --- | --- | --- | --- | --- | --- | --- | --- | --- | --- | --- | --- | --- | --- | --- | --- | --- | --- | --- | --- | --- | --- | --- | --- | --- | --- | --- | --- | --- | --- | --- | --- | --- | --- | --- | --- | --- | --- | --- | --- | --- | --- | --- | --- | --- | --- | --- | --- | --- | --- | --- | --- | --- | --- | --- | --- | --- | --- | --- | --- | --- | --- | --- | --- | --- | --- | --- | --- | --- | --- | --- | --- | --- | --- | --- | --- | --- | --- | --- | --- | --- | --- | --- | --- | --- | --- | --- | --- | --- | --- |

**S1.2: Search Strategy in Ovid Medline (05.12.2023)**

| \| 1. \| exp Randomized Controlled Trial/ \| \| --- \| --- \| \| 2. \| controlled clinical trial.pt. \| \| 3. \| randomized.ab,pt. \| \| 4. \| placebo.ab,pt. \| \| 5. \| drug therapy.fs. \| \| 6. \| randomly.ab. \| \| 7. \| trial.ab. \| \| 8. \| groups.ab. \| \| 9. \| 1 or 2 or 3 or 4 or 5 or 6 or 7 or 8 \| \| 10. \| exp animals/ not humans.sh. \| \| 11. \| ((Randomized Controlled Trial or controlled clinical trial or randomized or placebo or drug therapy or randomly or trial or groups) not (animals not humans)).sh. \| \| 12. \| exp Periodontal Diseases/ \| \| 13. \| exp Periodontitis/ \| \| 14. \| exp Alveolar Bone Loss/ \| \| 15. \| ((periodontal adj disease*) or periodontitis).mp. [mp=title, book title, abstract, original title, name of substance word, subject heading word, floating sub-heading word, keyword heading word, organism supplementary concept word, protocol supplementary concept word, rare disease supplementary concept word, unique identifier, synonyms, population supplementary concept word, anatomy supplementary concept word] \| \| 16. \| (periodont* adj2 pocket*).mp. [mp=title, book title, abstract, original title, name of substance word, subject heading word, floating sub-heading word, keyword heading word, organism supplementary concept word, protocol supplementary concept word, rare disease supplementary concept word, unique identifier, synonyms, population supplementary concept word, anatomy supplementary concept word] \| \| 17. \| (alveolar adj2 loss).mp. [mp=title, book title, abstract, original title, name of substance word, subject heading word, floating sub-heading word, keyword heading word, organism supplementary concept word, protocol supplementary concept word, rare disease supplementary concept word, unique identifier, synonyms, population supplementary concept word, anatomy supplementary concept word] \| \| 18. \| (alveolar adj2 atroph*).mp. [mp=title, book title, abstract, original title, name of substance word, subject heading word, floating sub-heading word, keyword heading word, organism supplementary concept word, protocol supplementary concept word, rare disease supplementary concept word, unique identifier, synonyms, population supplementary concept word, anatomy supplementary concept word] \| \| 19. \| (attachment adj3 loss).mp. [mp=title, book title, abstract, original title, name of substance word, subject heading word, floating sub-heading word, keyword heading word, organism supplementary concept word, protocol supplementary concept word, rare disease supplementary concept word, unique identifier, synonyms, population supplementary concept word, anatomy supplementary concept word] \| \| 20. \| exp Dental Scaling/ \| \| 21. \| exp Curettage/ \| \| 22. \| exp Subgingival Curettage/ \| \| 23. \| exp Debridement/ \| \| 24. \| exp Periodontal Debridement/ \| \| 25. \| ((non surgical adj4 therap*) or (nonsurgical adj4 therap*)).mp. [mp=title, book title, abstract, original title, name of substance word, subject heading word, floating sub-heading word, keyword heading word, organism supplementary concept word, protocol supplementary concept word, rare disease supplementary concept word, unique identifier, synonyms, population supplementary concept word, anatomy supplementary concept word] \| \| 26. \| ((non surgical adj4 treatment*) or (nonsurgical adj4 treatment*)).mp. [mp=title, book title, abstract, original title, name of substance word, subject heading word, floating sub-heading word, keyword heading word, organism supplementary concept word, protocol supplementary concept word, rare disease supplementary concept word, unique identifier, synonyms, population supplementary concept word, anatomy supplementary concept word] \| \| 27. \| ((non surgical adj4 care) or (nonsurgical adj4 care)).mp. [mp=title, book title, abstract, original title, name of substance word, subject heading word, floating sub-heading word, keyword heading word, organism supplementary concept word, protocol supplementary concept word, rare disease supplementary concept word, unique identifier, synonyms, population supplementary concept word, anatomy supplementary concept word] \| \| 28. \| ((non surgical adj4 maintenance) or (nonsurgical adj4 maintenance)).mp. [mp=title, book title, abstract, original title, name of substance word, subject heading word, floating sub-heading word, keyword heading word, organism supplementary concept word, protocol supplementary concept word, rare disease supplementary concept word, unique identifier, synonyms, population supplementary concept word, anatomy supplementary concept word] \| \| 29. \| (non surgical periodontal therap* or nonsurgical periodontal therap*).mp. [mp=title, book title, abstract, original title, name of substance word, subject heading word, floating sub-heading word, keyword heading word, organism supplementary concept word, protocol supplementary concept word, rare disease supplementary concept word, unique identifier, synonyms, population supplementary concept word, anatomy supplementary concept word] \| \| 30. \| NSPT.mp. \| \| 31. \| ((supragingival adj3 scal*) or (supra gingival adj3 scal*)).mp. [mp=title, book title, abstract, original title, name of substance word, subject heading word, floating sub-heading word, keyword heading word, organism supplementary concept word, protocol supplementary concept word, rare disease supplementary concept word, unique identifier, synonyms, population supplementary concept word, anatomy supplementary concept word] \| \| 32. \| ((supragingival adj3 debrid*) or (supra gingival adj3 debrid*)).mp. [mp=title, book title, abstract, original title, name of substance word, subject heading word, floating sub-heading word, keyword heading word, organism supplementary concept word, protocol supplementary concept word, rare disease supplementary concept word, unique identifier, synonyms, population supplementary concept word, anatomy supplementary concept word] \| \| 33. \| ((supragingival adj3 curett*) or (supra gingival adj3 curett*)).mp. [mp=title, book title, abstract, original title, name of substance word, subject heading word, floating sub-heading word, keyword heading word, organism supplementary concept word, protocol supplementary concept word, rare disease supplementary concept word, unique identifier, synonyms, population supplementary concept word, anatomy supplementary concept word] \| \| 34. \| (subgingival adj3 scal*).mp. [mp=title, book title, abstract, original title, name of substance word, subject heading word, floating sub-heading word, keyword heading word, organism supplementary concept word, protocol supplementary concept word, rare disease supplementary concept word, unique identifier, synonyms, population supplementary concept word, anatomy supplementary concept word] \| \| 35. \| (subgingival adj3 debrid*).mp. [mp=title, book title, abstract, original title, name of substance word, subject heading word, floating sub-heading word, keyword heading word, organism supplementary concept word, protocol supplementary concept word, rare disease supplementary concept word, unique identifier, synonyms, population supplementary concept word, anatomy supplementary concept word] \| \| 36. \| (subgingival adj3 curett*).mp. [mp=title, book title, abstract, original title, name of substance word, subject heading word, floating sub-heading word, keyword heading word, organism supplementary concept word, protocol supplementary concept word, rare disease supplementary concept word, unique identifier, synonyms, population supplementary concept word, anatomy supplementary concept word] \| \| 37. \| professional mechanical plaque removal.mp. \| \| 38. \| PMPR.mp. \| \| 39. \| (professional adj2 plaque removal).mp. [mp=title, book title, abstract, original title, name of substance word, subject heading word, floating sub-heading word, keyword heading word, organism supplementary concept word, protocol supplementary concept word, rare disease supplementary concept word, unique identifier, synonyms, population supplementary concept word, anatomy supplementary concept word] \| \| 40. \| (periodont* adj3 therap*).mp. [mp=title, book title, abstract, original title, name of substance word, subject heading word, floating sub-heading word, keyword heading word, organism supplementary concept word, protocol supplementary concept word, rare disease supplementary concept word, unique identifier, synonyms, population supplementary concept word, anatomy supplementary concept word] \| \| 41. \| (periodont* adj3 treatment*).mp. [mp=title, book title, abstract, original title, name of substance word, subject heading word, floating sub-heading word, keyword heading word, organism supplementary concept word, protocol supplementary concept word, rare disease supplementary concept word, unique identifier, synonyms, population supplementary concept word, anatomy supplementary concept word] \| \| 42. \| (periodont* adj3 care).mp. [mp=title, book title, abstract, original title, name of substance word, subject heading word, floating sub-heading word, keyword heading word, organism supplementary concept word, protocol supplementary concept word, rare disease supplementary concept word, unique identifier, synonyms, population supplementary concept word, anatomy supplementary concept word] \| \| 43. \| (periodont* adj3 maintenance).mp. [mp=title, book title, abstract, original title, name of substance word, subject heading word, floating sub-heading word, keyword heading word, organism supplementary concept word, protocol supplementary concept word, rare disease supplementary concept word, unique identifier, synonyms, population supplementary concept word, anatomy supplementary concept word] \| \| 44. \| supportive periodontal care.mp. \| \| 45. \| SPC.mp. \| \| 46. \| supportive periodontal treatment*.mp. \| \| 47. \| supportive periodontal therap*.mp. [mp=title, book title, abstract, original title, name of substance word, subject heading word, floating sub-heading word, keyword heading word, organism supplementary concept word, protocol supplementary concept word, rare disease supplementary concept word, unique identifier, synonyms, population supplementary concept word, anatomy supplementary concept word] \| \| 48. \| SPT.mp. [mp=title, book title, abstract, original title, name of substance word, subject heading word, floating sub-heading word, keyword heading word, organism supplementary concept word, protocol supplementary concept word, rare disease supplementary concept word, unique identifier, synonyms, population supplementary concept word, anatomy supplementary concept word] \| \| 49. \| (maintenance adj3 therap*).mp. [mp=title, book title, abstract, original title, name of substance word, subject heading word, floating sub-heading word, keyword heading word, organism supplementary concept word, protocol supplementary concept word, rare disease supplementary concept word, unique identifier, synonyms, population supplementary concept word, anatomy supplementary concept word] \| \| 50. \| (maintenance adj3 treatment*).mp. [mp=title, book title, abstract, original title, name of substance word, subject heading word, floating sub-heading word, keyword heading word, organism supplementary concept word, protocol supplementary concept word, rare disease supplementary concept word, unique identifier, synonyms, population supplementary concept word, anatomy supplementary concept word] \| \| 51. \| (maintenance adj3 care).mp. [mp=title, book title, abstract, original title, name of substance word, subject heading word, floating sub-heading word, keyword heading word, organism supplementary concept word, protocol supplementary concept word, rare disease supplementary concept word, unique identifier, synonyms, population supplementary concept word, anatomy supplementary concept word] \| \| 52. \| (maintenance adj3 recall*).mp. [mp=title, book title, abstract, original title, name of substance word, subject heading word, floating sub-heading word, keyword heading word, organism supplementary concept word, protocol supplementary concept word, rare disease supplementary concept word, unique identifier, synonyms, population supplementary concept word, anatomy supplementary concept word] \| \| 53. \| exp Follow-Up Studies/ \| \| 54. \| follow up.mp. \| \| 55. \| followup.mp. \| \| 56. \| 12 or 13 or 14 or 15 or 16 or 17 or 18 or 19 \| \| 57. \| 20 or 21 or 22 or 23 or 24 or 25 or 26 or 27 or 28 or 29 or 30 or 31 or 32 or 33 or 34 or 35 or 36 or 37 or 38 or 39 or 40 or 41 or 42 \| \| 58. \| 43 or 44 or 45 or 46 or 47 or 48 or 49 or 50 or 51 or 52 or 53 or 54 or 55 \| \| 59. \| 11 and 56 and 57 and 58 \| |
| --- | --- | --- | --- | --- | --- | --- | --- | --- | --- | --- | --- | --- | --- | --- | --- | --- | --- | --- | --- | --- | --- | --- | --- | --- | --- | --- | --- | --- | --- | --- | --- | --- | --- | --- | --- | --- | --- | --- | --- | --- | --- | --- | --- | --- | --- | --- | --- | --- | --- | --- | --- | --- | --- | --- | --- | --- | --- | --- | --- | --- | --- | --- | --- | --- | --- | --- | --- | --- | --- | --- | --- | --- | --- | --- | --- | --- | --- | --- | --- | --- | --- | --- | --- | --- | --- | --- | --- | --- | --- | --- | --- | --- | --- | --- | --- | --- | --- | --- | --- | --- | --- | --- | --- | --- | --- | --- | --- | --- | --- | --- | --- | --- | --- | --- | --- | --- | --- | --- |

**S1.3: Search Strategy on Cochrane Central Register of Controlled Trials (CENTRAL) (05.12.2023)**

| #1 | MeSH descriptor: [Periodontal Diseases] explode all trees |
| --- | --- |
| #2 | MeSH descriptor: [Alveolar Bone Loss] explode all trees |
| #3 | MeSH descriptor: [Periodontal Pocket] explode all trees |
| #4 | periodont* |
| #5 | periodont* NEAR/1 pocket* |
| #6 | periodont* NEAR/1 "attachment loss" |
| #7 | alveolar NEAR/1 loss |
| #8 | alveolar NEAR/1 atroph* |
| #9 | MeSH descriptor: [Dental Scaling] explode all trees |
| #10 | MeSH descriptor: [Curettage] explode all trees |
| #11 | MeSH descriptor: [Subgingival Curettage] explode all trees |
| #12 | MeSH descriptor: [Debridement] explode all trees |
| #13 | MeSH descriptor: [Periodontal Debridement] explode all trees |
| #14 | non-surgical NEAR/4 therap* |
| #15 | non-surgical NEAR/4 treatment* |
| #16 | non-surgical NEAR/4 care |
| #17 | non-surgical NEAR/4 maintenance |
| #18 | nonsurgical NEAR/4 therap* |
| #19 | nonsurgical NEAR/4 treatment* |
| #20 | nonsurgical NEAR/4 care |
| #21 | nonsurgical NEAR/4 maintenance |
| #22 | NSPT |
| #23 | supragingival NEAR/3 scal* |
| #24 | supragingival NEAR/3 curett* |
| #25 | supragingival NEAR/3 debrid* |
| #26 | supra-gingival NEAR/3 scal* |
| #27 | supra-gingival NEAR/3 curett* |
| #28 | supra-gingival NEAR/3 debrid* |
| #29 | subgingival NEAR/3 scal* |
| #30 | subgingival NEAR/3 curett* |
| #31 | subgingival NEAR/3 debrid* |
| #32 | sub-gingival NEAR/3 scal* |
| #33 | sub-gingival NEAR/3 curett* |
| #34 | subgingival NEAR/3 debrid* |
| #35 | root NEAR/3 plan* |
| #36 | professional NEAR/2 plaque removal |
| #37 | PMPR |
| #38 | periodont* NEAR/3 therap* |
| #39 | periodont* NEAR/3 treatment* |
| #40 | periodont* NEAR/3 care |
| #41 | MeSH descriptor: [Maintenance] explode all trees |
| #42 | periodont* NEAR/3 maintenance |
| #43 | SPT |
| #44 | SPC |
| #45 | maintenance NEAR/3 therap* |
| #46 | maintenance NEAR/3 treatment* |
| #47 | maintenance NEAR/3 care |
| #48 | maintenance NEAR/3 recall* |
| #49 | MeSH descriptor: [Follow-Up Studies] explode all trees |
| #50 | MeSH descriptor: [Aftercare] explode all trees |
| #51 | follow up |
| #52 | follow-up |
| #53 | followup |
| #54 | #1 OR #2 OR #3 OR #4 OR #5 OR #6 OR #7 OR #8 |
| #55 | #9 OR #10 OR #11 OR #12 OR #13 OR #14 OR #15 OR #16 OR #17 OR #18 OR #19 OR #20 OR #21 OR #22 OR #23 OR #24 OR #25 OR #26 OR #27 OR #28 OR #29 OR #30 OR #31 OR #32 OR #33 OR #34 OR #35 OR #36 OR #37 OR #38 OR #39 OR #40 |
| #56 | #41 OR #42 OR #43 OR #44 OR #45 OR #46 OR #47 OR #48 OR #49 OR #50 OR #51 OR #52 OR 53 |
| #57 | #54 AND #55 AND #56 |

**Supplemental Material 2: Excluded studies from second-stage screening**

| **Study Number** | **Author** | **Title** | **Year** | **Reasons for exclusion** |
| --- | --- | --- | --- | --- |
| 1 | Artzi Z.  Sudri S. | The impact of supportive periodontal treatment on the outcome of regenerative surgical therapy in aggressive periodontitis patients. | 2021 | Not a RCT |
| 2 | Caposio P.  Torta A.I.  Romano F.  Aimetti M.  Romagnoli R.  Marchiaro G.  Cirillo D.M. | Molecular approaches to the identification and treatment monitoring of periodontal pathogens. | 2003 | No follow-up ≥10 years, no tooth-level data of required parameters |
| 3 | ChiCTR-TRC-09000365, | A Study of the Relationship between Periodontitis and Systematic diseases and Therapic protocols | - | No follow-up ≥10 years |
| 4 | ChiCTR-TRC-10001062, | Effects of non-surgical periodontal treatment on clinical, immunological responses and metabolic control in type 2 diabetic patients: a randomized study | 2019 | No follow-up ≥10 years |
| 5 | ChiCTR-TRC-14004822, | The application and evaluation of Er, Cr: YSGG laser in the nonsugical treatment of periodontitis | 2020 | No follow-up ≥10 years |
| 6 | ChiCTR-TRC-14005088, | The effect of occlusal adjustment on the periodontium remodeling of anterior teeth with different attachment levels | 2019 | No test or control interventions in APT |
| 7 | Cortellini P.  Buti J.  Pini Prato G.  Tonetti M.S. | Periodontal regeneration versus extraction and dental implant or prosthetic replacement of teeth severely compromised by attachment loss to the apex: A randomized controlled clinical trial reporting 10-year outcomes, survival analysis and mean cumulative cost of recurrence. | 2017 | One of the two interventions involved extraction of teeth and primary outcome of systematic review is tooth loss and clinical parameters |
| 8 | Kaur H.  Grover V.  Malhotra R.  Gupta M. | Evaluation of curcumin gel as adjunct to scaling & root planing in management of periodontitis- randomized clinical & biochemical investigation. | 2019 | No follow-up ≥10 years |
| 9 | Neill M.E.  Mellonig J.T. | Clinical efficacy of the Nd:YAG laser for combination periodontitis therapy. | 1997 | No follow-up ≥10 years |
| 10 | Pietruska M.  Paniczko A.  Waszkiel D.  Pietruski J.  Bernaczyk A. | Efficacy of local treatment with chlorhexidine gluconate drugs on the clinical status of periodontium in chronic periodontitis patients. | 2006 | Not a RCT |
| 11 | RBR-8t4qqx, | Evaluation of the use of lactobacilli for gum treatment in patients with diabetes | - | No follow-up ≥10 years |
| 12 | Rieger L.  Carson R.E. | The clinical effects of saline and aloe vera rinses on periodontal surgical sites. | 2002 | No follow-up ≥10 years |
| 13 | Rosling B.  Hellstrom M.K.  Ramberg P.  Socransky S.S.  Lindhe J. | The use of PVP-iodine as an adjunct to non-surgical treatment of chronic periodontitis. | 2001 | Not a RCT |

**Supplemental Material 3: Meta-analyses of WMD in PPD between test and control groups at baseline, 1-year and 10-year endpoints (10-year follow-up studies)**

**Supplemental material 3a: WMD in PPD between test and control groups of included studies at baseline**


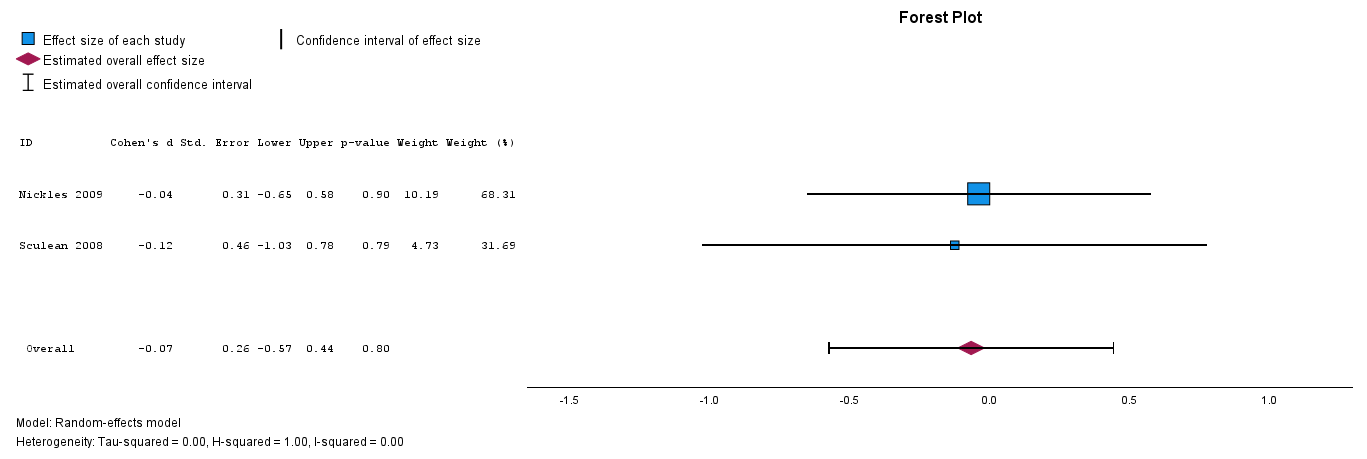


**Supplemental material 3b: WMD in PPD between test and control groups of included studies at 1-year**


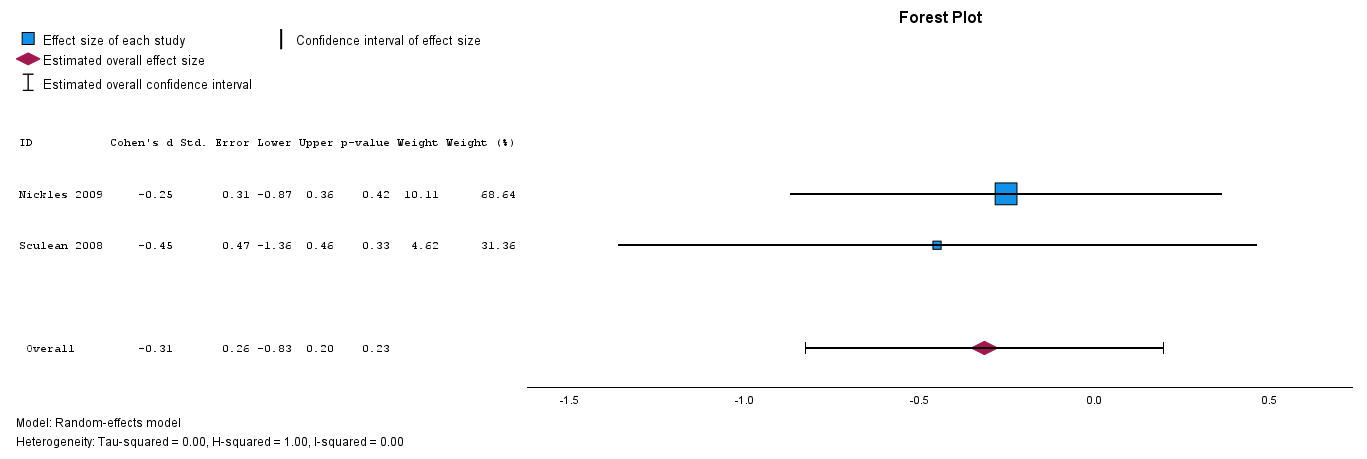


**Supplemental material 3c: WMD in PPD between test and control groups of included studies at respective endpoints (10-years)**


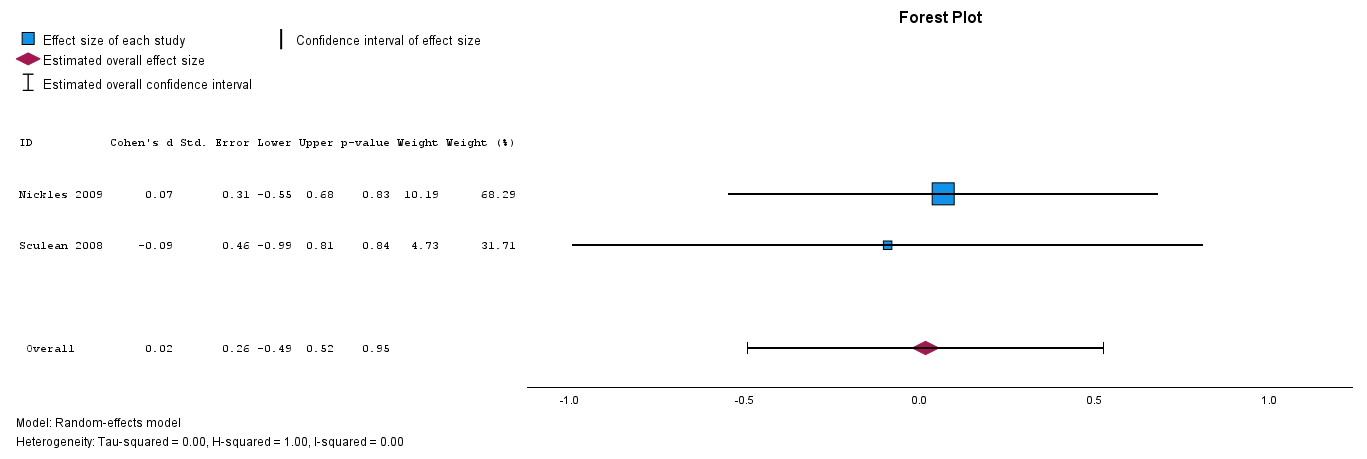


**Supplemental Material 4: Meta-analyses of WMD in CAL between test and control groups at baseline, 1-year and 10-year endpoints (10-year follow-up studies)**

**Supplemental material 4a: WMD in CAL between test and control groups of included studies at baseline**


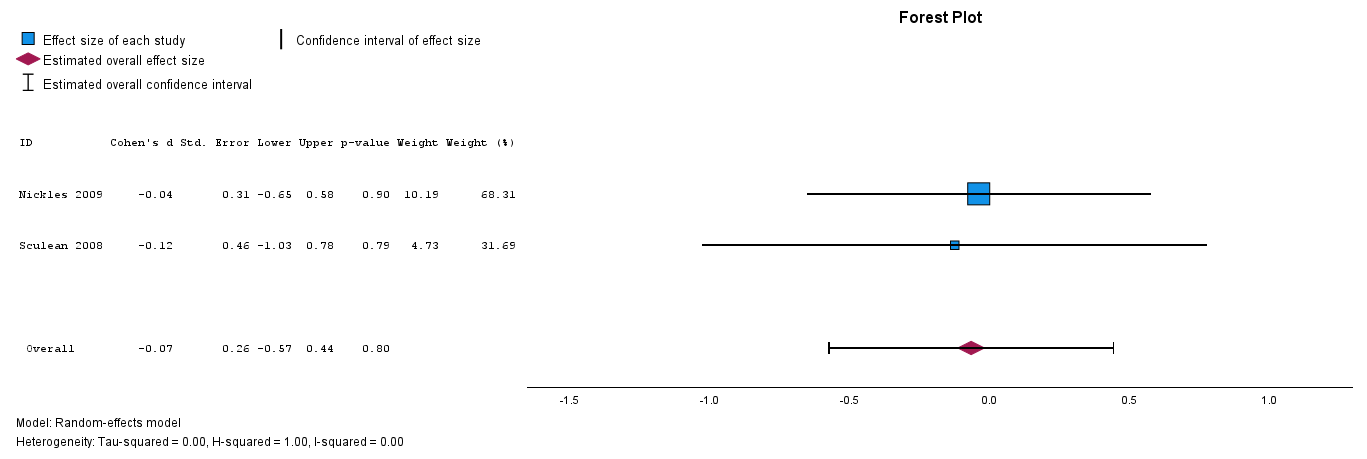


**Supplemental material 4b: WMD in CAL between test and control groups of included studies at 1-year**


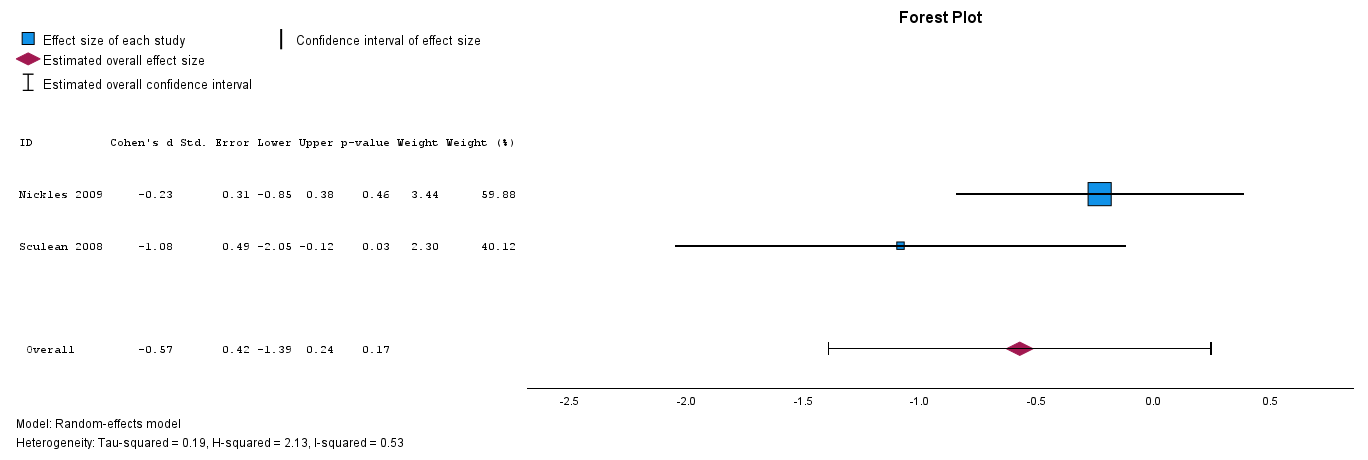


**Supplemental material 4c: WMD in CAL between test and control groups of included studies at respective endpoints (10-years)**


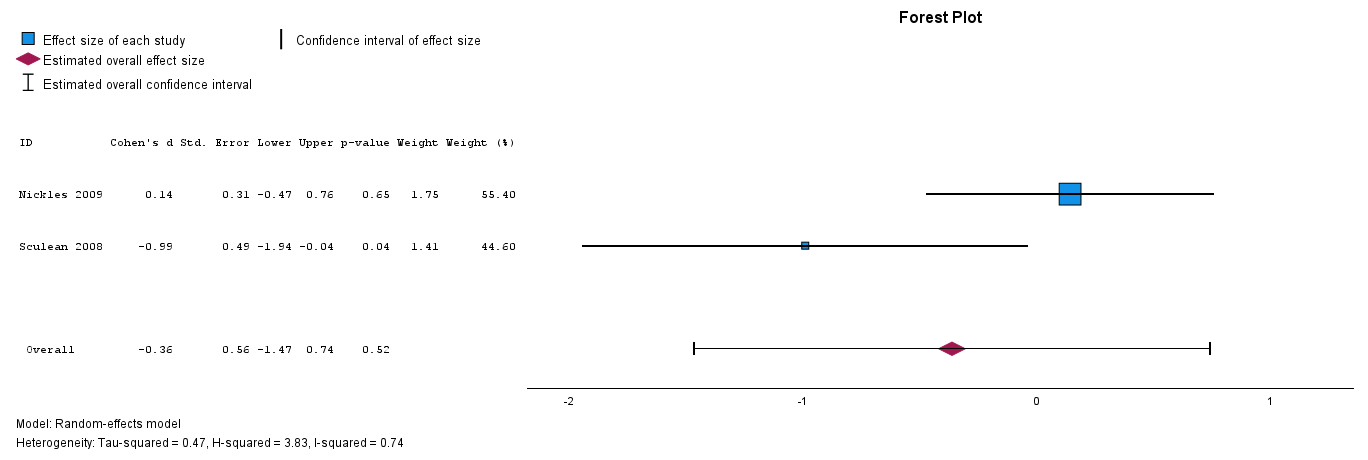


**Supplemental Material 5: Cochrane Risk of Bias 2 (RoB-2) assessments for RCTs**

| **Author** | **Randomisation** | **Deviation** | **Missing Data** | **Outcome Measurement** | **Selective Reporting** | **Overall Risk** |
| --- | --- | --- | --- | --- | --- | --- |
| Cortellini et al, 2017 | Low | Low | Low | High | Low | High |
| Nickles et al, 2009 | Low | Low | Low | High | Low | High |
| Petsos et al, 2019 | Some concerns | Low | High | Low | Low | High |
| Sculean et al, 2008 | Low | Low | Some concerns | High | Low | High |
| Cortellini et al, 2022 | Low | Low | High | Low | Low | High |
| Dori et al, 2013 | Low | Low | High | High | Low | High |
| Nygaard-Østby et al, 2010 | Low | Low | High | High | Low | High |
| Crespi et al, 2011 | Low | Low | Low | Low | Low | Low |
| Serino et al, 2001 | Low | Low | High | Low | Low | High |
